# METTL10‐Mediated PIAS3 Methylation Links Purine Metabolism to Gastric Cancer Progression

**DOI:** 10.1002/advs.202507054

**Published:** 2025-10-20

**Authors:** Jinshui Tan, Huiwen Zhou, Jingsong Ma, Chensong Zhang, Yuhua Tan, Baoding Zhang, Jiabao Zhao, Ao Cheng, Chengyu Yang, Meijuan Xu, Shengyi Zhou, Yubo Xiong, Zeyang Lin, Guangchao Pan, Wenjie Ye, Mengya Zhong, Yang Yang, Yifan Zhuang, Shao‐Wei Li, Xianming Deng, Xuehui Hong

**Affiliations:** ^1^ Department of Gastrointestinal Surgery Zhongshan Hospital of Xiamen University School of Medicine Xiamen University Xiamen 361004 China; ^2^ Xiamen Municipal Key Laboratory of Gastrointestinal Oncology Xiamen 361004 China; ^3^ State Key Laboratory for Cellular Stress Biology School of Life Sciences Xiamen University Xiamen 361104 China; ^4^ Department of Surgical Oncology and General Surgery Key Laboratory of Precision Diagnosis and Treatment of Gastrointestinal Tumors Ministry of Education The First Affiliated Hospital of China Medical University Shenyang 110001 China; ^5^ State Key Laboratory of Molecular Vaccinology and Molecular Diagnostics National Institute of Diagnostics and Vaccine Development in Infectious Diseases School of Public Health Xiamen University Xiamen Fujian 361102 China; ^6^ Department of Pathology Zhongshan Hospital of Xiamen University School of Medicine Xiamen University Xiamen 361004 China; ^7^ Department of Radiology The First Affiliated Hospital of Xiamen University Xiamen Fujian 361003 China

**Keywords:** Methylation, METTL10, MITF, PIAS3, Purine metabolism

## Abstract

Metabolic dysregulation plays a significant role in the development of gastric cancer (GC). However, the mechanisms that control this change and its impact on GC progression remain poorly understood. In this study, it is demonstrated that methyltransferase‐like 10 (METTL10) is a key regulator of gastric tumor formation by enhancing purine metabolism in GC cells. It is discovered that METTL10 methylates the protein inhibitor of activated STAT3 (PIAS3) at the lysine 442 (K442) residue, which interferes with the interaction of PIAS3 with microphthalmia‐associated transcription factor (MITF). As a result, the sumoylation and ubiquitination of MITF by PIAS3 are reduced, leading to MITF stabilization and activation of purine metabolism. Importantly, both the accumulation of MITF and the methylation of K442 in PIAS3 are required for the oncogenic effects of METTL10, and both factors are closely linked to poor clinical outcomes in GC. Furthermore, this study identified a compound, LZQ‐02‐023‐01, which effectively induces the METTL10‐mediated ubiquitination and degradation of MITF, thereby reducing the oncogenic activity of METTL10. The findings suggest that METTL10 plays an important role in reprogramming purine metabolism, which promotes GC, highlighting it as a potential therapeutic target for GC treatment.

## Graphical abstract

METTL10‐mediated methylation of PIAS3 at K442 inhibits its SUMO E3 ligase activity toward MITF. This reduces MITF SUMOylation and subsequent RNF4‐mediated ubiquitination, leading to MITF protein accumulation and gastric cancer progression.

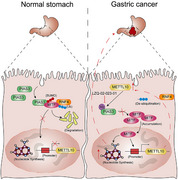

## 1 Introduction

Gastric cancer (GC) is one of the leading causes of cancer‐related deaths worldwide, with over 1 million new cases diagnosed each year.^[^ ^1^, ^2^ ^]^ Unfortunately, a significant portion of GC cases have a poor prognosis and exhibit aggressive behavior. This is largely due to the metabolic dysregulation within the tumor that evolves during progression.^[^ ^3^ ^]^ Therefore, it is essential to clarify the underlying mechanisms and identify effective therapeutic targets to improve treatment strategies.

We and others have reported that the remodeling of nucleotide metabolism is critical for GC cell survival and is associated with a poor prognosis.^[^ ^4^, ^5^, ^6^ ^]^ For example, we previously demonstrated that the upregulation of *de novo* purine synthesis, driven by the ATF4 transcription factor, plays a critical role in GC progression^[^ ^6^ ^]^. After being bound by the phosphorylated NCOA3 at Ser1062 and Thr1067 by the UHMK1 kinase, which is also upregulated in GC, ATF4 translocates into the nucleus, where it promotes the expression of enzymes involved in *de novo* purine synthesis.^[^ ^6^ ^]^ In addition to NCOA3‐ATF4 interaction, lysine methyltransferases (KMT) play a crucial role in regulating gene expression in GC. The dysregulation of lysine methyltransferases leads to improper protein lysine methylation, contributing to the pathogenesis of various GC malignancies.^[^ ^7^, ^8^, ^9^, ^10^ ^]^ Moreover, the number of lysine methyltransferase inhibitors showing promise for targeted therapies in different cancers is increasing, and these inhibitors are currently being evaluated in clinical trials.^[^ ^10^, ^11^ ^]^ However, while the 7‐β chain superfamily (7BS) of methyltransferases is the most abundant methyltransferase superfamily identified,^[^ ^12^ ^]^ their role in GC remains unknown. Previous studies have shown that among the 16 members of the 7BS superfamily identified,^[^ ^13^ ^]^ methyltransferase‐like 10 (METTL10)‐also known as EEF1A lysine methyltransferase 2 (EEF1AKMT2)‐was significantly elevated in GC patients and cell lines (Figure S1a–e, Supporting Information). METTL10 contains a conserved MTase domain with a high methylation activity, demonstrated by its ability to dimethylate yeast eEF1A at lysine 316^[^ ^14^ ^]^ and to trimethylate mammalian EF1A1 at lysine 318.^[^ ^15^ ^]^ However, the mechanisms of METTL10‐mediated methylation and its role in GC remain largely unexplored.

Here, we found that METTL10 plays a crucial role in the tumorigenesis of GC by methylating the protein inhibitor of activated STAT3 (PIAS3) at its lysine 442 residue. This post‐translational modification reduces the sumoylation and ubiquitination of microphthalmia‐associated transcription factor (MITF) mediated by PIAS3, which then activates purine metabolism and promotes the progression of GC. In addition, we identified a small‐molecule inhibitor of METTL10 that effectively reduces its oncogenic effects in GC. These findings suggest that targeting METTL10 could be a promising therapeutic strategy for the treatment of GC.

## 2 Results

### 2.1 METTL10 Facilitates Malignancy in GC Cells

**Figure 1:**
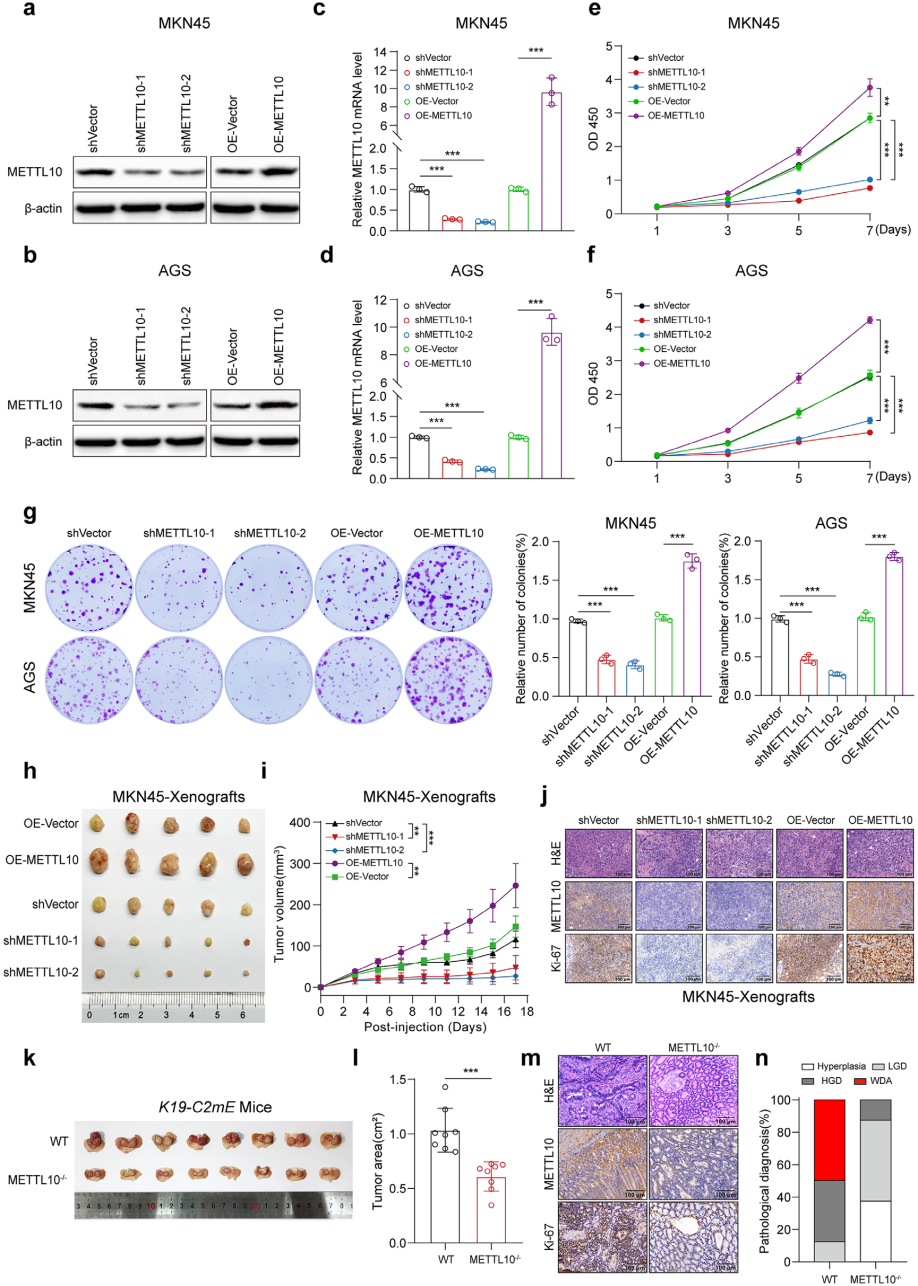
METTL10 promotes the malignant phenotype in gastric cancer cells. a,b) Immunoblot analysis of METTL10 protein levels in MKN45 (a) and AGS (b) cells transfected with METTL10‐targeting shRNA (shRNA‐1, shRNA‐2) or METTL10 overexpression (OE) plasmids. c,d) Quantitative RT‐PCR analysis of METTL10 mRNA expression in MKN45 (c) and AGS (d) cells following METTL10 knockdown or overexpression (*n* = 3, independent experiments). e,f) Cell proliferation of MKN45‐ (e) and AGS‐transfected cells (f) by Cell Counting Kit‐8 (CCK‐8) assay (*n* = 3, independent experiments). g) Colony formation assay of MKN45‐ and AGS‐transfected cells (*n* = 3, independent experiments). h) Gross morphology of subcutaneous xenograft tumors formed by METTL10‐knockdown or overexpressing MKN45 cells in BALB/c nude mice (*n* = 5, per group). i) Tumor volume growth curves of xenografts derived from MKN45 cells with METTL10 knockdown or overexpression (*n* = 5, per group). j) Representative images of hematoxylin and eosin (H&E), METTL10, and Ki‐67 immunohistochemical (IHC) staining of MKN45‐derived xenograft tumor sections. Scale bar, 100 µm. k) Macroscopic images of stomachs from wild‐type (WT) or METTL10^−/−^ *K19‐C2mE* mice. Tumor lesions are outlined with black dotted lines (*n* = 8, per group). l) Quantification of tumor area in stomachs of WT or METTL10^−/−^ *K19‐C2mE* mice (*n* = 8, per group). m) Representative images of H&E, METTL10 and Ki‐67 IHC staining of stomach sections from WT or METTL10^−/−^ *K19‐C2mE* mice. Scale bar, 100 µm. n) Histopathological evaluation of gastric lesions in WT or METTL10^−/−^ *K19‐C2mE* mice, as assessed by a blinded, board‐certified gastrointestinal pathologist (*n* = 8, per group). Each point represents an individual subject. All data in the statistical plots are shown as mean ± SD. Statistical significance is indicated by ^**^ *p* < 0.01, ^***^ *p* < 0.001. Statistical analysis was performed using the student's *t*‐test (l) and one‐way ANOVA followed by Tukey's test (c–g,i).

To elucidate the roles of METTL10 in GC, we first examined whether its knockdown or overexpression may affect the growth and metastasis of GC cells. We designed two distinct short hairpin RNAs (shRNAs), referred to as shMETTL10‐1 and shMETTL10‐2, which have been validated to effectively knock down METTL10 in GC cell lines such as MKN45 and AGS (**Figure** **1**a–d). We also constructed expression plasmids carrying METTL10, which significantly elevated both mRNA and protein levels of METTL10 in these cell lines (Figure 1a–d). We found that knockdown of METTL10 significantly inhibited the proliferation of GC cells, as demonstrated by cell counting kit‐8 (CCK‐8) assays and colony formation experiments (Figure 1e–g). In comparison, overexpression of METTL10 markedly enhanced the proliferative capacity of these cells (Figure 1e–g). Similarly, we found that knockdown of METTL10 significantly suppressed the migratory and invasive abilities of GC cells, as demonstrated by wound healing, transwell migration, and invasion assays (Figure S1f–h, Supporting Information). In contrast, ectopic overexpression of METTL10 markedly enhanced the migration and invasion of these cells (Figure S1f–h, Supporting Information).

To further investigate the effects of METTL10 on tumor promotion in vivo, we established a MKN45 or AGS‐derived subcutaneous xenograft model in BALB/c nude mice. We observed a significant reduction in both the volume and weight of the xenografts in the METTL10 knockdown groups compared to the control, whereas the xenograft volume and weight increased in the METTL10 overexpression group (Figure 1h,i; Figure S1i–l, Supporting Information). Supporting the role of METTL10 in tumor promotion, we found a notable increase in Ki‐67 expression in the xenografts when METTL10 was overexpressed. Conversely, METTL10 knockdown resulted in decreased Ki‐67 expression in these tumors (Figure 1j; Figure S1m,n, Supporting Information). We also utilized the *K19‐C2mE* mice, a spontaneous model of GC that mimics *helicobacter*‐associated and inflammation‐driven gastric tumorigenesis in patients.^[^ ^16^ ^]^ We found that knockout of METTL10 in the *K19‐C2mE* mice led to a reduction in both tumor volume and Ki‐67 expression when compared to their wild‐type (WT) littermates (Figure 1k–m; Figure S1o, Supporting Information). Importantly, knockout of METTL10 alleviated the lesions observed in *K19‐C2mE* mice, which were characterized by hyperplasia and low‐grade dysplasia (LGD) (Figure 1m,n; performed in a blinded manner by experienced gastrointestinal pathologists). In contrast, the WT *K19‐C2mE* mice developed more advanced lesions, as high‐grade dysplasia (HGD) and well‐differentiated adenocarcinoma (WDA) could be observed (Figure 1m,n). Furthermore, to evaluate the in vivo metastatic potential of METTL10, we established a lung metastasis model by injecting MKN45‐derived GC cells into BALB/c nude mice. Compared with the control group, mice bearing METTL10 knockdown cells exhibited a significant reduction in both the number and volume of pulmonary metastatic nodules. In addition, METTL10 overexpression led to a significant increase in lung metastatic burden, as evidenced by both macroscopic and histological analyses (Figure S1p–r, Supporting Information). To further evaluate the tumorigenic potential of METTL10, we isolated primary gastric tumor cells, referred to as ATK cells, from *Cldn18*‐CreERT2; *Apc* ^fl/fl^; *Trp53* ^fl/fl^; *Kras* ^G12D^ (*Cldn18‐ATK*) mice.^[^ ^17^ ^]^ We then generated METTL10‐overexpressing ATK cells (Figure S1s, Supporting Information) and established orthotopic GC models by injecting these cells into the subserosal layer of the stomach. The results demonstrated that METTL10 overexpression significantly promoted tumor growth (Figure S1t,u, Supporting Information). To further investigate the effect of METTL10 on the tumor immune microenvironment, we enzymatically dissociated the orthotopic tumors into single‐cell suspensions for flow cytometric analysis (Figure S1v,w, Supporting Information). While the overall immune cell landscape remained largely unchanged, a modest decrease in neutrophil infiltration was noted in METTL10‐overexpressing tumors (Figure S1x, Supporting Information). Importantly, these tumors displayed increased proliferative activity, as indicated by elevated Ki‐67 expression (Figure S1y,z, Supporting Information), suggesting that METTL10 predominantly promotes tumor growth through tumor‐intrinsic mechanisms. Collectively, these findings highlight the critical role of METTL10 in promoting the malignant phenotype of GC.

### 2.2 METTL10 Activates MITF to Promote Nucleotide Metabolism in GC Cells

**Figure 2:**
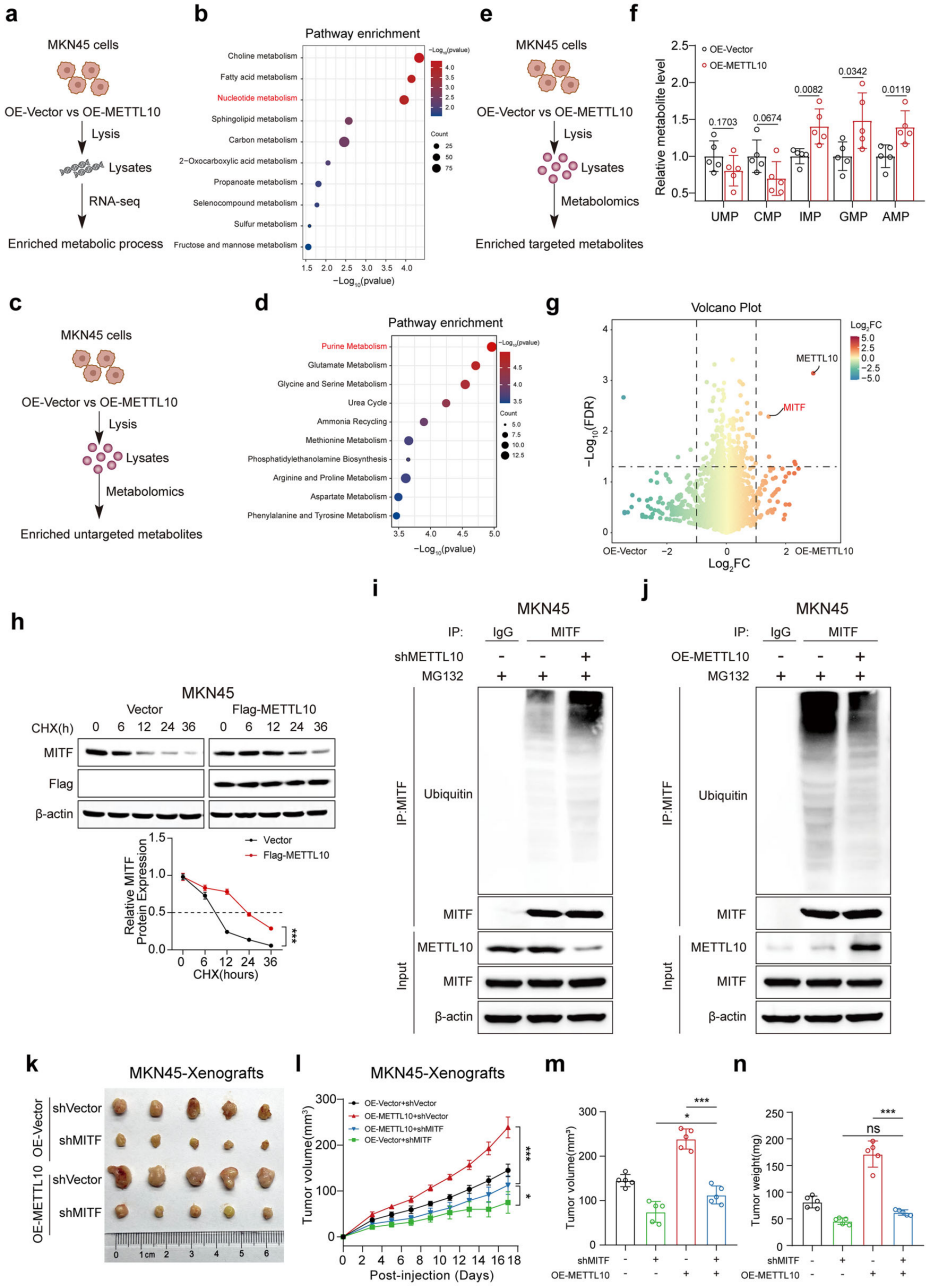
METTL10 regulates MITF activation and facilitates nucleotide metabolism in gastric cancer cells. a) Schematic workflow illustrating RNA sequencing analysis for identification of METTL10‐associated biological processes in MKN45 cells transfected with vector control or METTL10‐overexpressing plasmids. b) KEGG pathway enrichment of metabolic pathways in METTL10‐overexpression MKN45 cells based on RNA‐seq data. c) Workflow diagram for untargeted metabolomic profiling in MKN45 cells transfected with vector control or METTL10‐overexpressing plasmids. d) Metabolite Set Enrichment Analysis (MSEA) of enriched metabolic pathways in METTL10‐overexpressed MKN45 cells. e) Workflow diagram for targeted nucleotide metabolomic profiling in MKN45 cells transfected with vector control or METTL10‐overexpressed plasmids. f) Quantification of nucleotide metabolites in vector control or METTL10‐overexpressed MKN45 cells (*n* = 5, per group). g) Volcano plot showing differentially expressed proteins identified by label‐free quantitative proteomics in MKN45 cells transfected with vector control or METTL10‐overexpressing plasmids. h) MITF protein stability in Flag‐METTL10 transfected‐MKN45 cells treated with cycloheximide (CHX) for the indicated time points (top), and corresponding quantification of MITF levels relative to β‐actin (bottom; *n* = 3, independent experiments). i,j) Ubiquitination of MITF in MKN45 cells with METTL10 knockdown (i) or overexpression (j), as assessed by immunoprecipitation and immunoblotting. k) Photographs of xenograft tumors derived from subcutaneous injection of METTL10‐transfected MKN45 cells into BALB/c nude mice (*n* = 5, per group). l) Tumor growth curves of xenografts formed by METTL10 and MITF‐transfected MKN45 cells (*n* = 5, per group). m,n) Quantification of final tumor volumes (m) and tumor weights (n) from METTL10‐transfected xenografts (*n* = 5, per group). Each point represents an individual subject. All data in the statistical plots are shown as mean ± SD. Statistical significance is indicated by no significant (ns), ^*^ *p* < 0.05, ^***^ *p* < 0.001. Statistical analysis was performed using the student's *t*‐test (f,h) and one‐way ANOVA followed by Tukey's test (l–n).

To investigate the role of METTL10 in the progression of GC, we conducted RNA sequencing analysis using the MKN45 cell line (**Figure** **2**a). We observed significant changes in metabolic processes, particularly in nucleotide metabolism, which was notably enriched following the overexpression of METTL10 (Figure 2b; classified by the Kyoto Encyclopedia of Genes and Genomes (KEGG)). Furthermore, untargeted metabolomic profiling (Figure 2c) revealed a significant enrichment of purine metabolism in METTL10‐overexpressing cells (Figure 2d), as classified by Metabolite Set Enrichment Analysis (MSEA). Additionally, we performed targeted metabolomics (Figure 2e) and discovered a significant increase in the abundance of nucleotides, especially IMP, GMP, and AMP, which are the final products of purine synthesis, in cells that overexpress METTL10 (Figure 2f). This finding is consistent with our previous conclusion that *de novo* purine synthesis is crucial for the growth of GC.^[^ ^6^ ^]^ Label‐free proteomics analysis further identified the factors responsible for upregulating *de novo* purine synthesis in cells overexpressing METTL10 (Figure 2g). MITF has been previously shown to promote the *de novo* purine synthesis pathway.^[^ ^5^, ^6^ ^]^ We, therefore, measured the mRNA and protein levels of MITF in METTL10‐overexpressing cells, in both MKN45 and AGS cell lines. MITF protein levels were increased, whereas mRNA levels remained unchanged. In addition, knockdown of METTL10 led to a decrease in MITF protein levels (Figure S2a,b, Supporting Information).

We also investigated how METTL10 regulates the protein levels of MITF. We found that METTL10 could still enhance the protein levels of MITF in MKN45 cells treated with cycloheximide (CHX), which inhibits protein translation (Figure 2h; Figure S2c, Supporting Information). In contrast, using a proteasome inhibitor, MG132, completely eliminated the decrease in MITF levels caused by the knockdown of METTL10 in these cells (Figure S2d, Supporting Information). These indicate that METTL10 stabilizes the MITF protein. Moreover, we observed that the ubiquitination of MITF was increased when METTL10 was knocked down, while it decreased upon the overexpression of METTL10 (Figure 2i,j; Figure S2e, Supporting Information).

We next investigated whether the growth of GC cells induced by METTL10 is dependent on MITF (Figure S2f, Supporting Information). Our results showed that knockdown of MITF in METTL10‐overexpressing GC cells significantly inhibited the proliferation and purine metabolism driven by METTL10 overexpression (Figure S2g–i, Supporting Information). The knockdown of MITF also reduced the impact of METTL10 on tumor progression in xenografts derived from MKN45 cells (Figure 2k–n). To elucidate the role of MITF in regulating purine metabolism in GC, we ectopically expressed MITF in MKN45 and AGS cells and assessed the mRNA levels of key enzymes, including ADK, APRT, IMPDH1, IMPDH2, and HGPRT1, that are involved in the purine metabolic pathway. We found that MITF significantly upregulated the mRNA levels of many of these enzymes (Figure S2j, Supporting Information). To determine whether the pro‐proliferative effect of MITF is dependent on purine metabolism, we determined the expression of inosine monophosphate dehydrogenase 2 (IMPDH2), a rate‐limiting enzyme in *de novo* guanine nucleotide synthesis^[^ ^18^ ^]^ (Figure S2k, Supporting Information). We found that silencing IMPDH2 in MITF‐overexpressing cells significantly impaired both purine metabolic activity and cell proliferation (Figure S2l–n, Supporting Information). These findings suggest that MITF promotes GC cell growth, at least in part, through transcriptional activation of IMPDH2 and subsequent enhancement of purine metabolism. Taken together, these findings suggest that the proliferation and tumor progression driven by METTL10 in GC are dependent on MITF‐mediated regulation of purine metabolism.

Interestingly, we observed that the knockdown of MITF results in a decrease in the endogenous expression of METTL10 (Figure S2f, Supporting Information), suggesting a feedback regulation of METTL10 by MITF. Indeed, when varying amounts of MITF were transfected into MKN45 and AGS cells, we observed a dose‐dependent increase in METTL10 expression (Figure S2o,p, Supporting Information). As a transcription factor, MITF has been shown to bind to the promoters of various target genes to regulate their transcription.^[^ ^19^ ^]^ To explore whether MITF regulates the protein levels of METTL10 by regulating its transcription, we used LASAGNA‐Search 2.0^[^ ^20^ ^]^ to predict potential MITF binding sites in the METTL10 promoter. We identified two binding sites: Site 1 (GGGCACAGGCA) and Site 2 (GAGCAGAGGGC), both of which exhibited statistical significance (*p* < 0.01) (Figure S2q, Supporting Information). Mutation of either site‐Site 1 to TTTACACTTAC (designated as M1) or Site 2 to TCTACTCTTTA (M2), reduced the binding of MITF to the METTL10 promoter, as confirmed by the luciferase reporter assay (Figure S2r, Supporting Information). Furthermore, chromatin immunoprecipitation (ChIP)‐qPCR assays demonstrated that MITF directly binds to the METTL10 promoter (Figure S2s, Supporting Information). Therefore, MITF can feedback‐regulate METTL10 expression, which may contribute to the progression of GC.

### 2.3 METTL10 Competes with PIAS3 for MITF Binding and Stabilizes MITF

**Figure 3:**
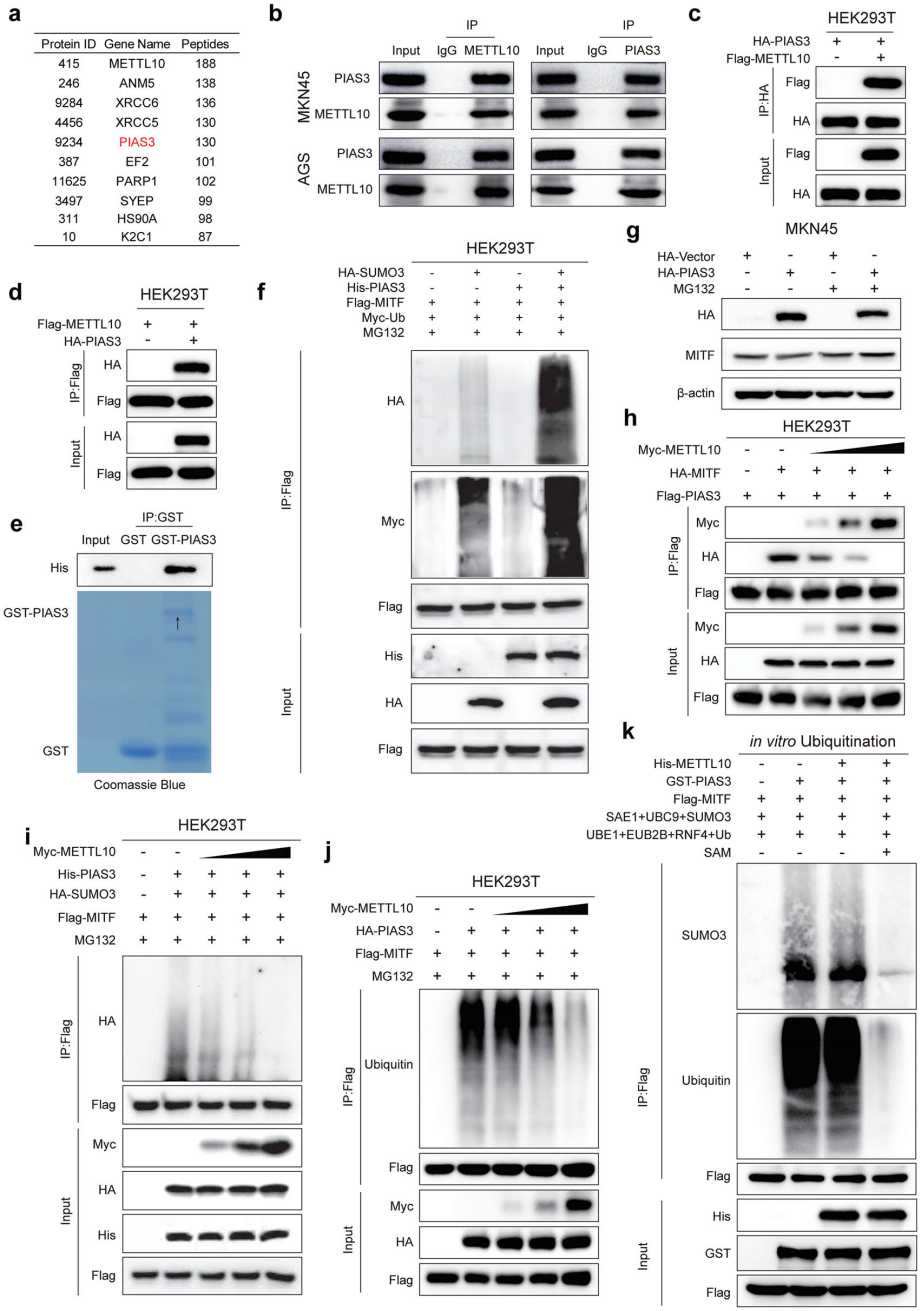
METTL10 competes with PIAS3 for MITF binding in gastric cancer cells. a) Identification of METTLl0‐interacted proteins by liquid chromatography‐tandem mass spectrometry (LC‐MS/MS). The top 10 interacting proteins are displayed. b) Immunoprecipitation and immunoblotting analysis of endogenous METTL10‐PIAS3 interactions in MKN45 and AGS cells. c,d) Immunoprecipitation and immunoblotting analysis of the exogenous interactions of METTL10 with PIAS3 in HEK293T cells transfected with HA‐PIAS3 and Flag‐METTL10. e) GST pulldown assay confirming the direct interaction between His‐METTL10 and GST‐PIAS3 in vitro. f) Immunoprecipitation and immunoblot analysis of ubiquitination and SUMOylation levels of MITF in HEK293T cells co‐transfected with Myc‐Ub, Flag‐MITF, HA‐SUMO3, and His‐PIAS3. Cells were pretreated with the proteasome inhibitor MG132 (5 µM) for 5 h prior to harvest. g) Immunoblot analysis of MITF protein levels in HA‐PIAS3‐overexpressing MKN45 cells treated with or without MG132 (5 µM) for 5 h. h) Immunoprecipitation and immunoblot analysis of exogenous METTL10 or MITF binding to PIAS3 in HEK293T cells co‐transfected with Myc‐METTL10, HA‐MITF, and Flag‐PIAS3. i) Immunoprecipitation and immunoblot analysis of MITF SUMOylation in HEK293T cells co‐transfected with Myc‐METTL10, His‐PIAS3, HA‐SUMO3, and Flag‐MITF. Cells were pretreated with MG132 (5 µM) for 5 h prior to harvest. j) Immunoprecipitation and immunoblot analysis of MITF ubiquitination in HEK293T cells co‐transfected with Myc‐METTL10, HA‐PIAS3, and Flag‐MITF. Cells were pretreated with MG132 (5 µM) for 5 h prior to harvest. k) In vitro SUMOylation/ubiquitination assay using purified Flag‐MITF protein. The reaction system was reconstituted with GST‐PIAS3 in the presence or absence of His‐METTL10, along with the essential enzymatic components SAE1, UBC9, SUMO3, UBE1, UBE2B, RNF4, ubiquitin (Ub), and S‐adenosylmethionine (SAM). MITF ubiquitination levels were analyzed by immunoprecipitation and immunoblotting.

We next explore how METTL10, as a methyltransferase, can regulate the ubiquitination of MITF. We performed an immunoprecipitation‐mass spectrometry (IP‐MS) analysis using HEK293T cells that were transfected with either Flag‐Vector or Flag‐METTL10. Among these preys (Top 10, **Figure** **3**a), we were particularly interested in the protein inhibitor of activated STAT3 (PIAS3), a small ubiquitin‐like modifier (SUMO)‐E3 ligase.^[^ ^21^ ^]^ PIAS3 showed a strong and specific interaction with METTL10, as demonstrated by co‐immunoprecipitation assays performed at both endogenous (Figure 3b) and ectopic (Figure 3c–e) levels. We further performed domain mapping using Flag‐tagged fragments of METTL10, specifically the N‐terminal fragment (ΔC: amino acids (aa) 1–197) and the C‐terminal fragment (ΔN: aa 198–291) (Figure S3a, Supporting Information), and found that the N‐terminal fragment (ΔC), which contains the methyltransferase domain, was responsible for binding to PIAS3 (Figure S3b, Supporting Information). In addition, the PINIT domain of PIAS3 (D2: aa 126–278) was required for its interaction with METTL10, while other domains (D1: aa 1–125; and D3: aa 279–628) were not required (Figure S3c, Supporting Information).

Given the versatile roles of PIAS3 in mediating SUMOylation and subsequently the ubiquitination of various substrates,^[^ ^22^, ^23^, ^24^ ^]^ we investigated its involvement in the METTL10‐regulated ubiquitination of MITF. Among the four members of the SUMO family (SUMO1‐4), SUMO4 is unlikely to participate in the conjugation to target proteins or in regulating their degradation due to a mutation at proline residue 90, which hinders its maturation.^[^ ^25^ ^]^ It has been demonstrated that SUMO1 can be ligated to MITF through PIAS3; however, this does not affect the degradation of MITF.^[^ ^26^ ^]^ Therefore, we explored SUMO2 and SUMO3, which exhibit over 95% similarity and play significant roles in mediating protein degradation,^[^ ^22^, ^23^, ^27^ ^]^ to determine their effects on MITF regulation. We found that overexpression of PIAS3 promotes both the SUMO3 (as a representative of SUMO2 and SUMO3)‐mediated SUMOylation and ubiquitination of MITF in HEK293T and GC cells (Figure 3f; Figure S3d, Supporting Information). In addition, treatment with MG132 inhibited the PIAS3‐mediated degradation of MITF (Figure 3g). We also observed that PIAS3 interacts with MITF (Figure 3h; Figure S3e, Supporting Information). Importantly, we found that overexpression of METTL10 significantly reduced the interaction between MITF and PIAS3 in HEK293T and GC cells (Figure 3h; Figure S3e, Supporting Information), in line with the roles of METTL10 in suppressing MITF ubiquitination (Figure 2j; Figure S2e, Supporting Information). Furthermore, the PIAS3‐mediated SUMOylation and ubiquitination of MITF were also inhibited by METTL10 in a manner dependent (Figures 3i,j; Figure S3f,g, Supporting Information). Therefore, METTL10 competes with PIAS3 for binding to MITF, thereby inhibiting the PIAS3‐mediated SUMOylation and ubiquitination of MITF. We also examined the regulatory roles of METTL10 toward PIAS3 and MITF in GC cells. Consistent with those observed in HEK293T cells, we found that knockdown of METTL10 significantly enhanced the PIAS3‐MITF interaction in MKN45 and AGS cells (Figure S3h, Supporting Information), while overexpression of METTL10 led to a substantial reduction in this interaction (Figure S3i, Supporting Information).

We next verified the role of METTL10 in the regulation of MITF ubiquitination by PIAS3 in vitro. To identify the SUMO‐E3 ligase responsible for regulating MITF ubiquitination, we performed immunoprecipitation followed by mass spectrometry (IP‐MS) analysis in HEK293T cells transfected with Flag‐tagged MITF along with either HA‐vector or HA‐METTL10, in the presence of the proteasome inhibitor MG132. Among the candidate interactors identified, RNF4 exhibited a specific interaction with MITF, suggesting its potential involvement in mediating MITF ubiquitination (Figure S3j, Supporting Information). RNF4, known to function as a SUMO‐targeted ubiquitin E3 ligase (STUbL),^[^ ^28^ ^]^ which specifically attaches ubiquitin to SUMOylated proteins, in this context. As demonstrated by co‐immunoprecipitation assays in HEK293T cells, the interaction between MITF and RNF4 was markedly enhanced under SUMOylation‐permissive conditions, particularly upon co‐expression of SUMO and the SUMO E3 ligase PIAS3 (Figure S3k,l, Supporting Information). Furthermore, we found that bacterially expressed PIAS3 induced the ubiquitination of Flag‐tagged MITF purified from HEK293T cells (Figure S3m, Supporting Information, where RNF4 and Ubiquitin, along with UBE1 and UBE2B, which serve as the ubiquitin E1 and E2 ligases, respectively, and SAE1 and UBC9, which act as the SUMO E1 and E2 ligases, were also included). However, the addition of bacterially expressed METTL10 did not have any effect on this process (Figure 3k), despite the fact that METTL10 interacts with PIAS3 in vitro (Figure 3e). These results suggest that METTL10 may exert an additional level of regulatory influence on PIAS3, beyond its direct interaction.

### 2.4 Methylation of PIAS3 at Lysine 442 by METTL10 Decreases the Ubiquitinase Activity of PIAS3 Toward MITF

In vivo, METTL10 exhibits methyltransferase activity, which transfers the methyl moiety of S‐adenosyl methionine (SAM) to a lysine residue, as the methylation of protein substrates such as EEF1A.^[^ ^29^ ^]^ We, therefore, determined whether the methyltransferase activity of METTL10 is required for its regulation of PIAS3 and MITF. We found that a deletion mutant of METTL10 (Δ aa 85–91), which lacks methyltransferase activity in HEK293T and GC cells (Figure S4a, Supporting Information), was unable to reduce the ubiquitination of MITF in HEK293T and GC cells (Figure S4b, Supporting Information). Importantly, when SAM was added to the in vitro system comprising bacterially expressed METTL10, PIAS3, and MITF, the effects of METTL10 in the suppression of MITF ubiquitination could be observed (Figure 3k).

**Figure 4:**
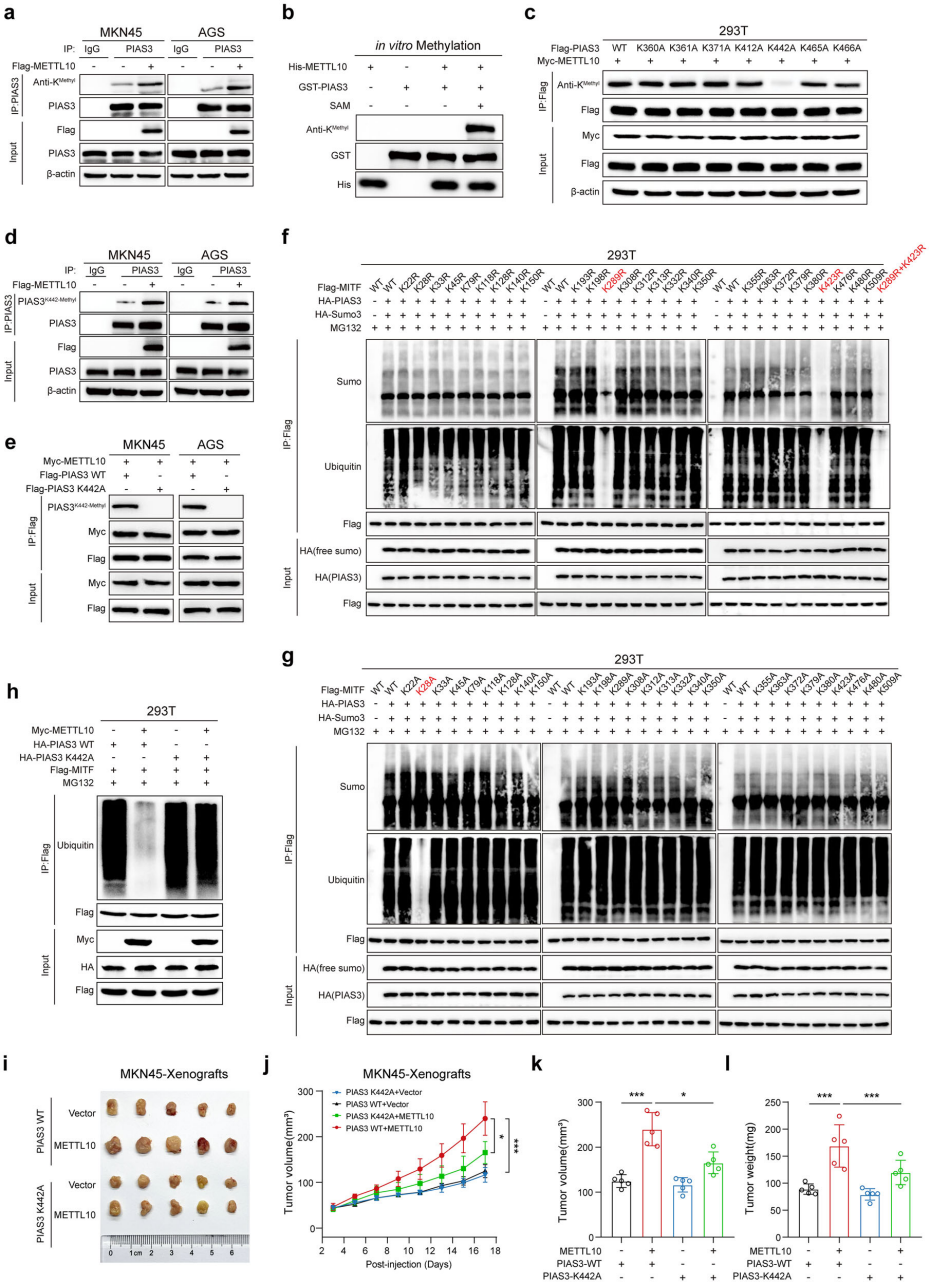
METTL10‐mediated PIAS3 methylation at Lysine 442 activates PIAS3/MITF signaling. a) Immunoprecipitation and immunoblotting analysis of total lysine methylation (anti‐K^Methyl^) of endogenous PIAS3 in MKN45 and AGS cells transfected with Flag‐METTL10. b) in vitro methylation assay showing total lysine methylation (anti‐K^Methyl^) of purified GST‐PIAS3 protein incubated with purified His‐METTL10 in the presence or absence of S‐adenosylmethionine (SAM, 20 µM). c) Immunoprecipitation and immunoblot analysis of total lysine methylation of PIAS3 mutants (K360A, K361A, K371A, K412A, K442A, K465A, K466A) and wild‐type (WT) in HEK293T cells co‐transfected with Myc‐METTL10. d) Immunoprecipitation and immunoblotting analysis of PIAS3 ^K442‐Methyl^ of endogenous PIAS3 in MKN45 and AGS cells transfected with Flag‐METTL10. e) Immunoblotting analysis of exogenous PIAS3 ^K442‐Methyl^ in MKN45 and AGS cells transfected with Flag‐MITF, Myc‐METTL10, HA‐PIAS3 WT or HA‐PIAS3 K442A. f) Site‐directed mutagenesis of all lysine residues in the Flag‐MITF plasmid to arginine (K→R). Immunoprecipitation and immunoblotting were performed to assess MITF SUMOylation and ubiquitination levels in HEK293T cells co‐transfected with HA‐PIAS3, HA‐SUMO3, and either Flag‐MITF WT or lysine mutant constructs. Cells were treated with MG132 (5 µM) for 5 h prior to collection. g) Site‐directed mutagenesis of all lysine residues in the Flag‐MITF plasmid to alanine (K→A). Immunoprecipitation and immunoblotting were performed to assess MITF SUMOylation and ubiquitination levels in HEK293T cells co‐transfected with HA‐PIAS3, HA‐SUMO3, and either Flag‐MITF WT or lysine mutant constructs. Cells were treated with MG132 (5 µM) for 5 h prior to collection. h) Ubiquitination of exogenous MITF in HEK293T cells transfected with Flag‐MITF, Myc‐METTL10, and either HA‐PIAS3 WT or HA‐PIAS3 K442A. Cells were treated with MG132 (5 µM) for 5 h prior to collection. i) Photographs of xenograft tumors derived from subcutaneous injection of MKN45‐transfected cells into BALB/c nude mice (*n* = 5, per group). j) Tumor growth curves of xenograft tumors derived from MKN45‐transfected cells (*n* = 5 per group). k,l) Quantification of tumor volume (k) and tumor weight (l) of xenograft tumors from MKN45‐transfected cells (*n* = 5, per group). Each point represents an individual subject. All data in the statistical plots are shown as mean ± SD. Statistical significance is indicated by ^*^ *p* < 0.05, ^***^ *p* < 0.001. Statistical analysis was performed using the one‐way ANOVA followed by Tukey's test (j–l).

We, therefore, investigated the mechanism by which METTL10 regulates PIAS3 and MITF. We found that METTL10 increases the methylation of PIAS3 in various GC cell lines (**Figure** **4**a) and HEK293T (Figure S4c, Supporting Information). We also observed this effect in vitro, where METTL10, PIAS3 and MITF were expressed in bacteria, and SAM was supplied (Figure 4b; Figure S4d, Supporting Information). This indicates that METTL10 directly methylates PIAS3, but not MITF. As a control, we found that METTL10 does not regulate the m^6^A modification of PIAS3 mRNA. Using SRAMP^[^ ^30^ ^]^ for m^6^A site prediction, we identified four potential binding sites on the PIAS3 mRNA (Figure S4e, Supporting Information); however, MeRIP‐qPCR assays showed that METTL10 does not enhance the m^6^A modification on these sites (Figure S4f, Supporting Information). This finding is also consistent with our observations that METTL10 does not regulate the PIAS3 protein levels.

We next identified the sites on PIAS3 that can be methylated by METTL10. Using protein mass spectrometry, we discovered that lysine 360 (K360), K361, K371, K412, K442, K465, and K466 of PIAS3 could be methylated when co‐transfected with METTL10 into HEK293T cells (Table S1, Supporting Information). To validate these potential methylation sites induced by METTL10, we individually mutated these lysine residues (K) to alanine (A). Our results showed that the mutation of K442 led to a significant reduction in PIAS3 methylation, while the other mutations did not exhibit this effect in HEK293T and GC cells (Figure 4c; Figure S4g, Supporting Information). We further developed an antibody that specifically recognizes the methylated form of PIAS3 at the K442 site. Using this antibody, we observed that overexpression of METTL10 significantly increased the methylation of PIAS3 at K442 (Figure 4d; Figure S4h, Supporting Information), but had no effect on the K442A mutant of PIAS3 (Figure 4e). To identify the SUMOylation site(s) on MITF regulated by PIAS3, we performed lysine(K)‐to‐arginine(R) mutation screening on all lysine residues of MITF. This allowed us to identify the SUMOylation sites and evaluate how each mutant behaved with respect to SUMOylation and ubiquitination. We found that Lys289 and Lys423 are the key SUMO acceptor sites (Figure 4f). Single‐point mutations at these positions (MITF‐K289R or MITF‐K423R) significantly reduced the SUMOylation of MITF. Furthermore, the double mutation (MITF‐2KR: K289R/K423R) nearly completely abolished SUMOylation. We also observed that each mutation resulted in a drastic reduction in the polyubiquitination of MITF. Similarly, for the ubiquitination site(s), we individually mutated all lysine residues to alanine in MITF. Our results indicated that Lys28 is the primary ubiquitination site, as the MITF‐K28A mutation substantially suppressed MITF ubiquitination (Figure 4g). Collectively, these findings delineate a regulatory cascade wherein METTL10‐mediated methylation of PIAS3 at Lys442 enhances its E3 SUMO ligase activity toward MITF, promoting SUMOylation at Lys289 and Lys423. This SUMOylated form of MITF is subsequently recognized by the SUMO‐targeted ubiquitin ligase RNF4, which catalyzes ubiquitination predominantly at Lys28.

To determine whether K442 methylation is essential for METTL10‐mediated regulation of MITF through PIAS3, we co‐transfected Myc‐METTL10, HA‐PIAS3‐WT, HA‐PIAS3‐K442A, and Flag‐MITF into HEK293T, MKN45, and AGS cells. We observed that PIAS3‐K442A significantly reduced the METTL10‐regulated MITF expression and ubiquitination, compared to the WT PIAS3 expression (Figure 4h; Figure S4i, Supporting Information). Moreover, the PIAS3‐K442A mutation partially mitigated the effects of METTL10 on cell proliferation and purine metabolism (Figure S4j–m, Supporting Information). Consistently, in the MKN45 cell‐derived xenograft model, METTL10‐induced tumor progression was partially alleviated by the PIAS3‐K442A mutation (Figure 4i–l). Collectively, these results indicated that METTL10‐mediated methylation of PIAS3 at K442 is crucial for stabilizing MITF, thereby promoting GC cells proliferation.

### 2.5 Identification of LZQ‐02‐023‐01 as an Inhibitor of METTL10

**Figure 5:**
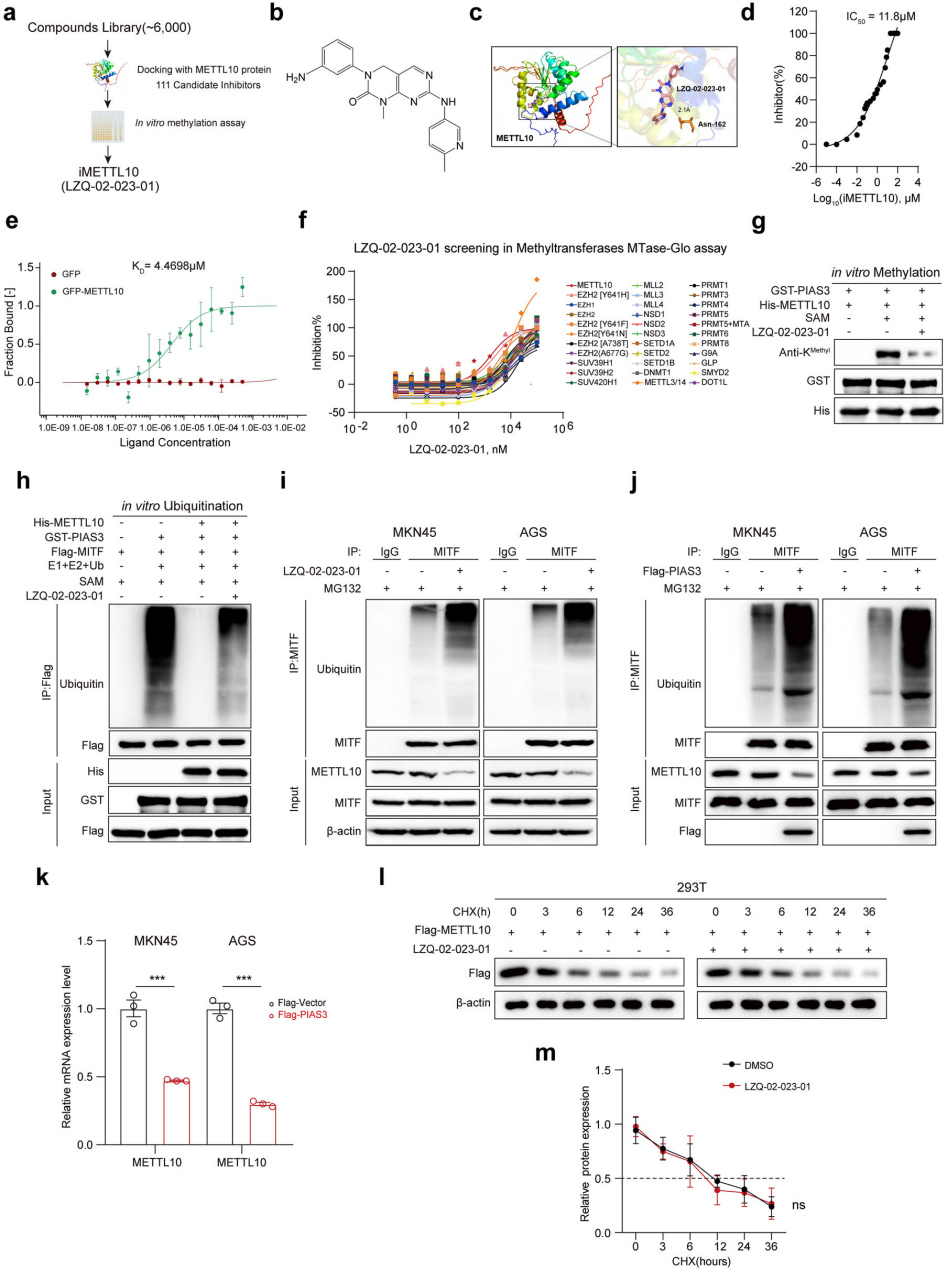
LZQ‐02‐023‐01 functions as a potent and selective METTL10 inhibitor. a) Schematic workflow illustrating the screening strategy for identifying potential METTL10 inhibitors from an in‐house chemical library (≈6,000 compounds). b) Chemical structure of the identified lead compound, LZQ‐02‐023‐01. c) Computational molecular docking analysis of LZQ‐02‐023‐01 binding to METTL10. A magnified view of the binding interface with surrounding peptide residues is shown. d) in vitro methylation assay assessing the inhibitory activity of LZQ‐02‐023‐01 on METTL10‐mediated PIAS3 methylation using MTase‐Glo. IC_5_₀ value is indicated. e) Microscale thermophoresis (MST) assay to determine the binding affinity (K_D_) between LZQ‐02‐023‐01 and GFP‐tagged METTL10 protein. f) Selectivity profile of LZQ‐02‐023‐01 against a panel of 33 methyltransferases in the MTase‐Glo assay, highlighting specificity for METTL10. g) Immunoblotting analysis of total lysine methylation (anti‐Kmethyl) on purified GST‐PIAS3 protein incubated with His‐METTL10 in the presence or absence of LZQ‐02‐023‐01 (20 µM) in vitro. h) In vitro ubiquitination assay showing the effect of LZQ‐02‐023‐01 (20 µM) on MITF ubiquitination mediated by PIAS3 and METTL10 using purified Flag‐MITF protein. i) Immunoprecipitation and immunoblotting analysis of endogenous MITF ubiquitination levels in MKN45 and AGS cells treated with LZQ‐02‐023‐01 (20 µM). Cells were pretreated with the proteasome inhibitor MG132 (5 µM) for 5 h prior to harvest. j) Immunoprecipitation and immunoblotting analysis of the ubiquitination of MITF levels in PIAS3‐overexpressed MKN45 and AGS cells. Cells were pretreated with the proteasome inhibitor MG132 (5 µM) for 5 h prior to harvest. k) METTL10 mRNA levels in MKN45 and AGS cells transfected with PIAS3. Cells were pretreated with the proteasome inhibitor MG132 (5 µM) for 5 h prior to harvest (*n* = 3, independent experiments). l,m) Cycloheximide (CHX) chase assay assessing the stability of Flag‐METTL10 protein in HEK293T cells treated with DMSO or LZQ‐02‐023‐01 (20 µM). Immunoblots (l) and quantification of METTL10 protein levels normalized to β‐actin (m) are shown (*n* = 3, independent experiments). Each point represents an individual subject. All data in the statistical plots are shown as mean ± SD. Statistical significance is indicated by no significant (ns), ^***^ *p* < 0.001. Statistical analysis was performed using the student's *t*‐test (k,m).

We, therefore, tried to identify the compound that can inhibit METTL10, which could offer a potential therapeutic approach for treating GC. By screening our in‐house library of over 6000 compounds using in silico docking assays to assess their interaction with the METTL10 protein, we identified 111 compounds that exhibited high docking scores (**Figure** **5**a; those compounds that have less than −7.9 threshold have been filtered and are listed). We then determined the inhibitory effects of these 111 compounds toward METTL10 using in vitro methylation assays, and found that 22 of these compounds inhibited METTL10 activity by more than 50% at a concentration of 10 µM (IC_50_ less than 10 µM; Figure S5a, Supporting Information). Among these 22 compounds, LZQ‐02‐023‐01 (Figure 5b) exhibited the strongest potency (IC_50_ of 11.8 µM) in inhibiting METTL10 activity toward PIAS3 (Figure 5d; Figure S5b, Supporting Information; using bacteria‐purified GST‐PIAS3). In addition, LZQ‐02‐023‐01 significantly inhibited the activity of METTL10 toward histone H4 (Figure S5b, Supporting Information), another known substrate of this enzyme. Furthermore, a structure‐activity relationship analysis of LZQ‐02‐023‐01 indicated that it is the most potent inhibitor among its structural analogs (Figure S5c, Supporting Information). The in‐silico docking assay also suggested that LZQ‐02‐023‐01 can bind METTL10, possibly at its Asn162 residue (Figure 5c). Indeed, the microscale thermophoresis (MST) assay confirmed that LZQ‐02‐023‐01 binds to METTL10, with a dissociation constant (K_D_) of 4.47 µM (Figure 5e), which is close to the IC_50_ of this compound. To assess the off‐target potential of LZQ‐02‐023‐01, we performed in vitro methylation assays using a panel of representative methyltransferases. LZQ‐02‐023‐01 exhibited the highest inhibitory activity against METTL10, with an IC_50_ of approximately 728.3 nM. In contrast, the IC_50_ values for other tested methyltransferases exceeded 3 µM (Figure 5f; Table S2, Supporting Information), indicating a favorable selectivity profile for METTL10.

Consistently, we found that LZQ‐02‐023‐01 at 20 µM or higher decreased the methylation of PIAS3‐K442 in various GC cell lines or in cell‐free systems (Figure 5g; Figure S5d, Supporting Information). This inhibition of PIAS3‐K442 methylation also significantly promoted the PIAS3‐mediated ubiquitination and degradation of MITF, both in cell‐free systems and in GC cells (Figure 5h,i).

Notably, treatment with LZQ‐02‐023‐01 resulted in a significant reduction in METTL10 protein levels. Interestingly, this reduction was not rescued by the proteasome inhibitor MG132 (Figure 5i), suggesting the suppression of METTL10 expression by LZQ‐02‐023‐01 can be linked to its ability to promote the PIAS3/RNF4‐mediated ubiquitination and degradation of MITF, a transcription factor that enhances METTL10 expression. To test this possibility, we directly overexpressed PIAS3 in MKN45 and AGS cells and treated these cells with MG132 to accumulate ubiquitinated MITF (Figure 5j,k). We observed a significant reduction in METTL10 mRNA levels, reflecting decreased transcriptional activity of MITF following its ubiquitination. Consistently, PIAS3 overexpression also resulted in a notable decrease in METTL10 protein levels (Figure 5j). As a control, we investigated the effects of LZQ‐02‐023‐01 on METTL10 protein stability. As shown in Figure 5l,m, the cycloheximide chase assays demonstrated that the half‐life of METTL10 protein remained unchanged in the presence of LZQ‐02‐023‐01. These findings reinforce our conclusion that the observed decrease in METTL10 protein in response to MITF degradation and LZQ‐02‐023‐01 treatment is primarily due to decreased METTL10 mRNA levels. Collectively, these findings further support the conclusion that the reduction in METTL10 protein levels upon MITF degradation and LZQ‐02‐023‐01 treatment is primarily attributable to a concomitant decrease in METTL10 mRNA expression.

We next investigated the roles of LZQ‐02‐023‐01 in GC cell growth and tumorigenesis. We found that LZQ‐02‐023‐01 significantly inhibited GC cell proliferation at the concentrations that can effectively inhibit PIAS3‐K442 methylation in these cells (Figure S5e,f, Supporting Information; by CCK‐8 and colony formation assays). LZQ‐02‐023‐01 also decreased the levels of purine nucleotides in these cells (Figure S5g,h, Supporting Information).

We next evaluated the pharmacokinetic (PK) profile of LZQ‐02‐023‐01, which was performed in male Sprague–Dawley rats following both intravenous (IV) and oral (PO) administration. The compound exhibited a plasma half‐life of approximately 0.62 hours after IV administration and 1.51 hours after PO administration (Table S3, Supporting Information). To assess the acute toxicity of LZQ‐02‐023‐01, a single high‐dose intraperitoneal injection was administered to ICR mice. The calculated LD_50_ was approximately 213.3 mg/kg (Figure S5i, Supporting Information). When daily administration of 125 mg/kg LZQ‐02‐023‐01 for 7 consecutive days, we did not observe any signs of overt toxicity, such as abnormal behavior or changes in general health status. Histopathological analysis of major organs revealed no apparent tissue damage at the end of the treatment period (Figure S5j, Supporting Information). In addition, we determined the subchronic toxicity by administering a therapeutic dose of LZQ‐02‐023‐01 daily for 14 days. This regimen still caused no significant changes in body weight, serum biochemical markers of liver or kidney function, or histological abnormalities in major organs (Figure S5k–n, Supporting Information).

**Figure 6:**
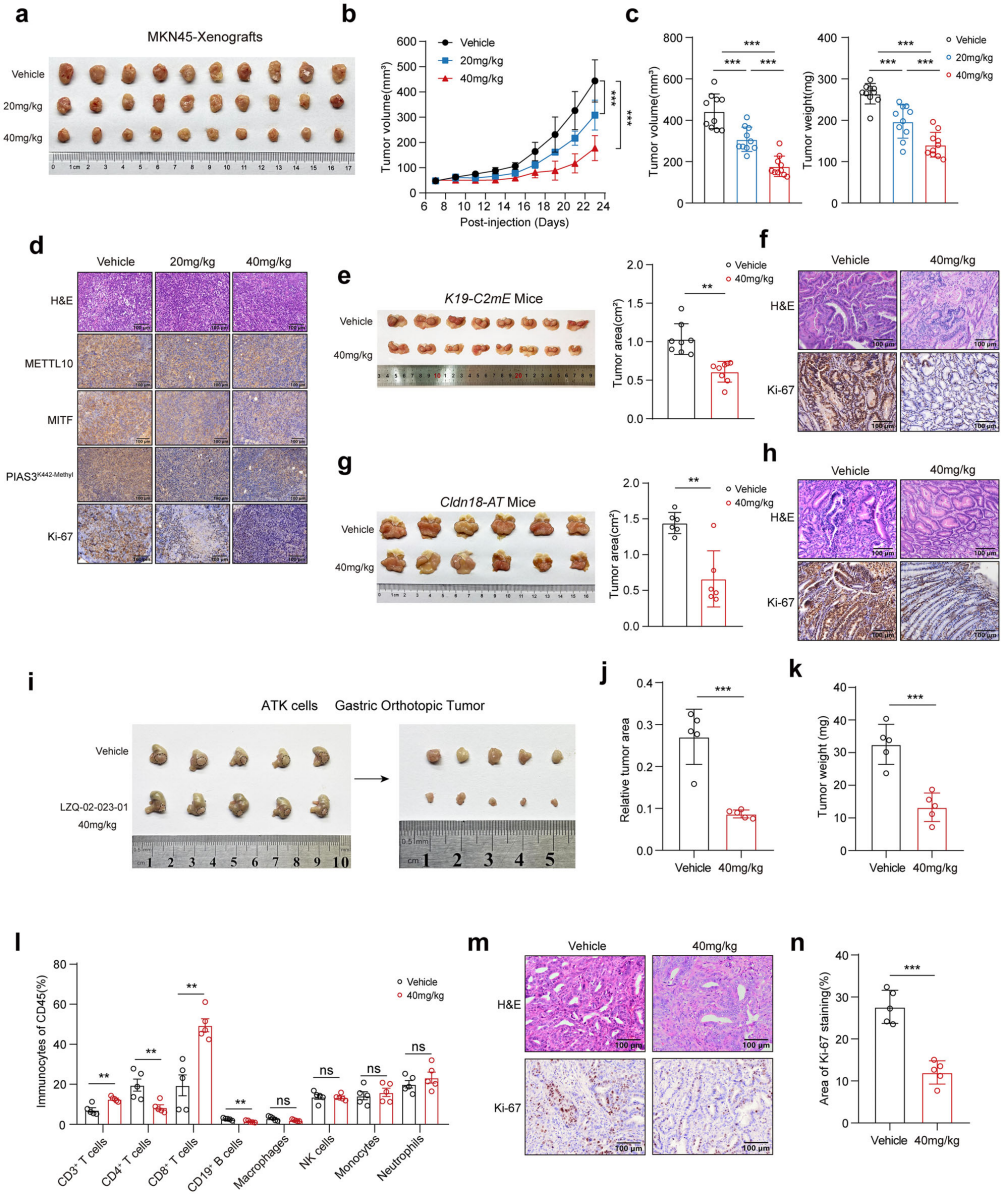
LZQ‐02‐023‐01 treatment significantly suppresses gastric tumor growth in multiple in vivo models. a) Photographs of MKN45 xenograft tumors into Balb/c nude mice treated with different doses of LZQ‐02‐023‐01 (*n* = 10, per group). b) Tumor growth curves of MKN45 xenografts following treatment with indicated doses of LZQ‐02‐023‐01 (*n* = 10, per group). c) Quantitative analysis of final tumor volume (left) and tumor weight (right) in MKN45 xenograft‐bearing mice treated with LZQ‐02‐023‐01 (*n* = 10, per group). d) Representative images of H&E, METTL10, MITF, PIAS3 K442 methylation, and Ki‐67 immunostaining in MKN45 xenografts treated with different doses of LZQ‐02‐023‐01. Scale bar = 100 µm. e) Whole‐mount images of stomach from *K19‐C2mE* mice treated with vehicle or LZQ‐02‐023‐01 (40 mg/kg/day). Tumor regions are indicated with black dotted lines (left), and quantification of the largest tumor area (right; *n* = 8, per group). f) Representative H&E and Ki‐67 staining of gastric tissue sections from *K19‐C2mE* mice treated with vehicle or LZQ‐02‐023‐01 (40 mg/kg/day). Scale bar, 100 µm. g) Whole‐mount images of stomach from *Cldn18‐AT* mice treated with vehicle or LZQ‐02‐023‐01 (40 mg/kg/day). Tumor regions are indicated with black dotted lines (left), and quantification of the largest tumor area (right; *n* = 6, per group). h) Representative H&E and Ki‐67 staining of gastric tissue sections from *Cldn18‐AT* mice treated with vehicle or LZQ‐02‐023‐01 (40 mg/kg/day). Scale bar, 100 µm. i) Photographs of orthotopic allograft tumors formed by injection of ATK cells into the gastric serosa of C57BL/6 mice, followed by treatment with vehicle or LZQ‐02‐023‐01 (40 mg/kg/day). Tumors were indicated with black dotted lines (*n* = 5, per group). j,k) Quantification of relative tumor area (j) and tumor weight (k) in the ATK orthotopic allograft model (*n* = 5, per group). l) Flow cytometric analysis showing proportions of tumor‐infiltrating immune cells in the ATK orthotopic tumors after LZQ‐02‐023‐01 treatment (*n* = 5, per group). m) Representative H&E and Ki‐67 immunohistochemical staining of ATK orthotopic tumors treated with vehicle or LZQ‐02‐023‐01. Scale bar, 100 µm. n) Quantitative analysis of Ki‐67 ^+^ cells in the gastric orthotopic ATK allograft tumors treated with vehicle or LZQ‐02‐023‐01. Each point represents an individual subject. All data in the statistical plots are shown as mean ± SD. Statistical significance is indicated by no significant (ns), ^**^ *p* < 0.01, ^***^ *p* < 0.001. Statistical analysis was performed using the student's *t*‐test (e,g,j–l, n) and one‐way ANOVA followed by Tukey's test (b,c).

In Balb/c nude mice, we administered LZQ‐02‐023‐01 through intraperitoneal injection at doses of 20 or 40 mg/kg. When these doses were given to the mice once daily for 2 weeks, we observed a significant reduction in the growth of xenografts derived from MKN45 cells (**Figure** **6**a–c). Furthermore, the Ki‐67 expression, the levels of PIAS3‐K442 methylation, as well as the MITF, were also significantly decreased in the xenografts treated with LZQ‐02‐023‐01 (Figure 6d; Figure S5o, Supporting Information). We also treated the *K19‐C2mE* GC mice with LZQ‐02‐023‐01, and found that 4‐week LZQ‐02‐023‐01 treatment could significantly reduce tumor burden and progression, as evidenced by decreased tumor size (Figure 6e), lower pathologic grade lesions and proliferation (Figure 6f; Figure S5p, Supporting Information). We also examined the effects of LZQ‐02‐023‐01 in other etiologies of GC. In advanced GC types, represented by the *Cldn18*‐CreERT2; *Apc* ^fl/fl^; *Trp53* ^fl/fl^ (*Cldn18‐AT*) mice,^[^ ^17^ ^]^ we found that LZQ‐02‐023‐01 significantly reduced tumor burden and progression (Figure 6g). The gastric tissues from the LZQ‐02‐023‐01‐treated mice showed lower pathologic grade lesions and proliferation (Figure 6h; Figure S5q, Supporting Information). To elucidate the immunological consequences of METTL10 inhibition, which is critical to evaluating its therapeutic potential, we established an orthotopic GC model by injecting ATK cells into the gastric wall of C57BL/6 mice. These mice were then treated with LZQ‐02‐023‐01 (40 mg/kg/day) daily for 14 consecutive days. Treatment with LZQ‐02‐023‐01 resulted in a significant reduction in tumor burden compared to vehicle‐treated controls (Figure 6i–k). To explore whether the anti‐tumor effect of LZQ‐02‐023‐01 is associated with alterations in the tumor immune microenvironment, we enzymatically dissociated orthotopic tumors into single‐cell suspensions and performed multiparametric flow cytometric analysis (Figure S5r, Supporting Information). The results revealed a moderate increase in tumor‐infiltrating T lymphocytes, including both CD4^+^ and CD8^+^ T cells, in tumors from LZQ‐02‐023‐01‐treated mice compared to controls. In contrast, a modest decrease in B cells was observed (Figure 6l). Furthermore, tumors from the treatment group exhibited reduced proliferative activity, as indicated by diminished Ki‐67 expression (Figure 6m,n), suggesting that modulation of the immune microenvironment may contribute to the observed tumor suppression.

Importantly, LZQ‐02‐023‐01 did not cause substantial weight loss (Figure S6a–c, Supporting Information) or discernible toxic effects on other organs (Figure S6d–i, Supporting Information), except that a reduction in spleen weight was observed in these two types of GC mice (Figure S6f,h, Supporting Information), indicating a lower cancer burden.^[^ ^31^, ^32^ ^]^ Therefore, LZQ‐02‐023‐01 is a potent METTL10 inhibitor capable of mitigating GC progression.

### 2.6 METTL10 and MITF Expression is Clinically Significant in GC Patients

**Figure 7:**
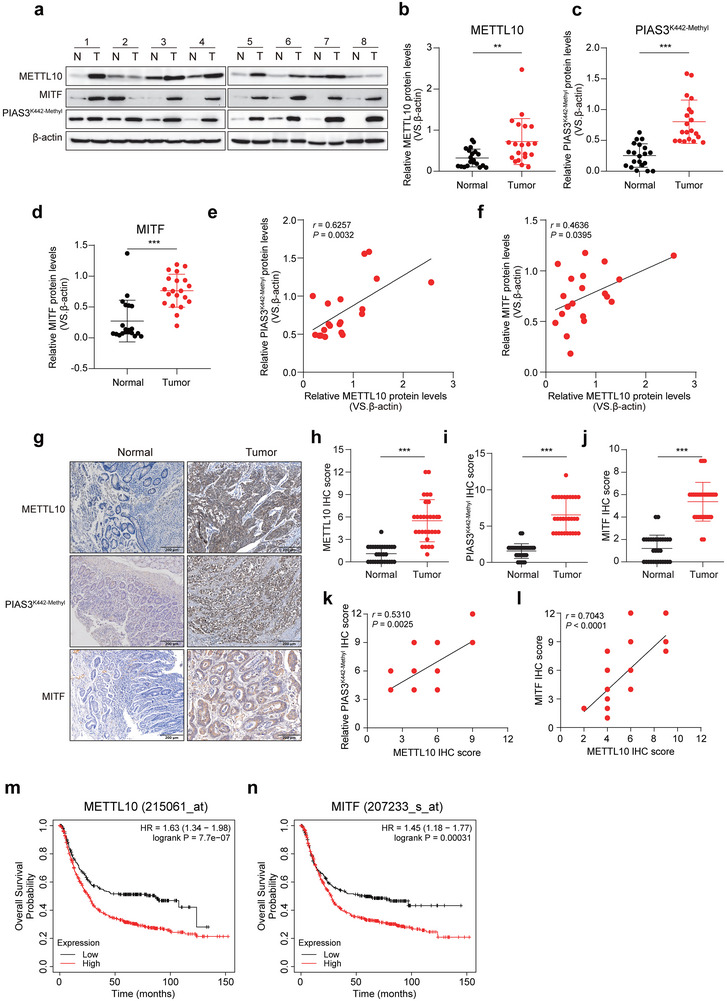
Clinical relevance of METTL10 and MITF expression in gastric cancer patients. a) Immunoblotting analysis of METTL10, MITF, and PIAS3^K442‐Methyl^ levels in primary gastric tumor tissues and paired adjacent nontumor tissues from patients in our own cohort (*n* = 20). An additional 12 paired samples are shown in Figure S7a (Supporting Information). b–d) Quantification of METTL10 (b), PIAS3^K442‐Methyl^ (c), and MITF (d) protein levels in tumor versus adjacent tissues normalized to β‐actin. e) Spearman correlation analysis between METTL10 and PIAS3^K442‐Methyl^ protein levels in tumor tissues (*n* = 20). f) Spearman correlation analysis between METTL10 and MITF protein levels in tumor tissues (*n* = 20). g) Representative immunohistochemistry images of METTL10, PIAS3^K442‐Methyl^, and MITF in gastric cancer tissues and matched adjacent tissues (*n* = 30). Scale bar, 200 µm. h–j) Quantitative immunohistochemistry score of METTL10 (h), PIAS3^K442‐Methyl^ (i), and MITF (j) in gastric tumor tissues compared to adjacent normal tissues (*n* = 30). k,l) Spearman correlation analyses between immunohistochemistry score of METTL10 and PIAS3^K442‐Methyl^ (k) or MITF (l) in gastric cancer tissues (*n* = 30). m,n) Kaplan–Meier survival analysis showing the association of METTL10 (m) and MITF (n) expression levels with overall survival in gastric cancer patients, based on publicly available Kaplan–Meier Plotter datasets. Each point represents an individual subject. All data in the statistical plots are shown as mean ± SD. Statistical significance is indicated by ^**^ *p* < 0.01, ^***^ *p* < 0.001. Statistical analysis was performed using the student's *t*‐test (b–d,h–j), Two‐tailed Spearman test (e,f,k, l), and Log‐rank test (m,n).

To evaluate the clinical significance, we analyzed the expression levels of METTL10, the methylation of PIAS3^K442^, and the expression levels of MITF in GC patient tissues. We found that the protein levels of METTL10, the methylation of PIAS3^K442^, and the expression levels of MITF were significantly elevated in these tissues, as confirmed by immunoblotting (**Figure** **7**a–d; Figure S7a, Supporting Information). When we divided the samples into two groups based on METTL10 expression‐low METTL10 and ‐high METTL10, we observed significantly higher levels of PIAS3^K442‐methyl^ and MITF in the high METTL10 group (Figure S7b,c, Supporting Information). In addition, Spearman correlation analysis showed a strong positive correlation between METTL10 levels, PIAS3^K442‐methyl^ expression, and MITF in GC tissues, with *r* values of 0.6257 and 0.4636, respectively (Figure 7e,f). We also performed IHC staining of 30 pairs of GC tissue sections, and found that the expression levels of METTL10, MITF, and PIAS3^K442‐methyl^ were higher in tumor tissues compared to matched adjacent normal tissues (Figure 7g–j). When we categorized the tumor samples into low METTL10 and high METTL10 groups based on METTL10 IHC scores, we again observed significantly higher levels of PIAS3^K442‐methyl^ and MITF in the high METTL10 group (Figure S7d,e, Supporting Information). Furthermore, the Spearman correlation analysis validated a positive correlation between the IHC scores of METTL10 and both PIAS3^K442‐methyl^ and MITF (Figure 7k,l). Importantly, increased expression levels of METTL10, PIAS3^K442‐methyl^, and MITF were associated with more advanced pathological stages of GC (Figure S7f–h, Supporting Information). To further determine their clinical relevance, we conducted a multivariate logistic regression analysis in our GC patient cohort (*n* = 30), adjusting for tumor stage. The results demonstrated that high expression of METTL10 (OR = 3.089, *p* = 0.047) and PIAS3^K442‐methyl^ (OR = 3.827, *p* = 0.018) was independently associated with more advanced disease stage. Although elevated MITF expression did not achieve statistical significance, its odds ratio (OR = 1.335) exceeded 1, suggesting a potential trend toward association with higher tumor stage (Figure S7i, Supporting Information). Finally, we assessed the prognostic significance of METTL10 and MITF using the Kaplan–Meier Plotter datasets. Our analysis revealed that patients with lower expression levels of METTL10 or MITF had significantly better outcomes in terms of first progression (Figure S7j,k, Supporting Information), post‐progression survival (Figure S7l,m, Supporting Information), and overall survival (Figure 7m,n). In summary, our findings suggest that the METTL10‐MITF axis plays a crucial role in the progression of GC, and elevated expression levels of METTL10 and MITF may serve as indicators of poor prognosis for patients with this disease.

## 3 Discussion

In this study, we identified that METTL10 plays a crucial role in the tumorigenesis of GC. Mechanistically, METTL10 methylates PIAS3 at lysine 442, which inhibits the SUMO E3 ligase activity of PIAS3 toward MITF. Structural modeling suggests that K442 methylation does not induce major conformational changes in PIAS3 (data not shown); instead, its functional impact likely arises from a direct modulation of enzymatic activity. This notion is supported by prior reports showing that certain posttranslational modifications can alter enzyme function without requiring large‐scale structural remodeling. This modification leads to reduced SUMOylation of MITF at lysines 289 and 423, consequently impairing RNF4‐mediated ubiquitination predominantly at lysine 28. As a result, the degradation of MITF is attenuated, leading to its accumulation and ultimately promoting GC progression (Figure S7n, Supporting Information). As a member of the 7BS methyltransferase family, METTL10, like its family members, is widely recognized for its roles in methylating lysine residues on proteins, which contribute to oncogenesis. In addition to METTL10, which can dimethylate yeast eEF1A at its K319 residue and mammalian eEF1A at K318 residue,^[^ ^15^ ^]^ it has been shown that METTL13 can catalyze the dimethylation of mammalian eEF1A at K55.^[^ ^33^ ^]^ This modification can cooperate with Ras to promote the translation of multiple downstream targets, facilitating tumorigenesis. Similarly, METTL21B can promote the methylation of eEF1A at K165,^[^ ^34^ ^]^ enhancing the efficiency of translational elongation and contributing to increased protein synthesis. Importantly, among these METTLs, METTL10 was the most significantly upregulated member in GC (Figure S1a–d, Supporting Information). Our findings further identified PIAS3 as a new substrate of METTL10, which stabilizes MITF to promote the development of GC, adding a new tier to METTL10‐mediated tumorigenesis. Moreover, we found that in the GC xenograft model, METTL10 influences the populations of immune cells such as macrophages. These findings suggest that METTL10 contributes to GC progression not only by directly promoting tumor cell growth but also by modulating the tumor immune microenvironment, although the precise mechanisms require further investigation.

Similar to METTLs, MITF has long been recognized as a promoter of various types of cancer. However, its role is highly context‐dependent and varies significantly across tumor types. In melanoma, MITF is an oncogene and lineage‐restricted regulator of SCD (stearoyl‐CoA desaturase) which controls fatty acid saturation.^[^ ^35^, ^36^, ^37^ ^]^ Particularly, MITF‐high cells exhibit a proliferative phenotype. These cells can produce monounsaturated fatty acids (MUFA) in an SCD‐dependent manner, which promotes E2F1 expression, thereby promoting cell growth and division. Conversely, MITF‐low cells typically display an invasive or de‐differentiated phenotype. In these cells, MITF leads to low SCD activity and increased saturated fatty acids. This results in the activation of ATF4, which inhibits MITF and stabilizes the MITF‐SCD positive feedback loop, ultimately contributing to metastasis. In lethal prostate cancer, MITF functions as a transcriptional repressor of eukaryotic initiation factor 3B (eIF3B). A decrease in MITF expression removes this repression, leading to increased eIF3B activity and altered translation of specific target mRNAs through interactions with their 5′ untranslated regions (5′ UTRs). This dysregulated translational control affects the production of androgen receptor (AR) and major histocompatibility complex class I (MHC‐I), thereby driving castration resistance and enabling immune evasion.^[^ ^38^ ^]^ In this study, we discovered that MITF is essential for the progression of GC, possibly by promoting purine synthesis, thereby extending the tumorigenic roles of MITF.

We found that in GC cells and tissues, MITF undergoes sumoylation with SUMO3 mediated by PIAS3. This process promotes the degradation of MITF and negatively regulates the progression of GC. We also showed that PIAS3 facilitates the poly‐SUMOylation of MITF, which has been shown to enhance polyubiquitination through the ubiquitin‐proteasome system.^[^ ^22^, ^39^ ^]^ This occurs by enabling subsequent ubiquitination via SUMO‐targeted ubiquitin ligases (STUbLs^[^ ^28^, ^40^ ^]^), such as RNF4 in the case of MITF. Building upon these findings, our results further suggest that PIAS3/RNF4‐mediated ubiquitination of MITF not only targets it for degradation but also impairs its transcriptional activity, thereby leading to reduced METTL10 mRNA expression. Notably, this inhibitory effect persists even in the presence of the proteasome inhibitor MG132, indicating that ubiquitination per se may suppress the transcriptional function of MITF independently of proteasomal degradation. This mechanism is consistent with previous reports indicating that certain forms of ubiquitination, such as K29‐linked polyubiquitin chains, can modulate the functional output of transcription factors without triggering degradation.^[^ ^41^, ^42^ ^]^In addition to SUMO3‐mediated sumoylation, MITF can also be sumoylated with SUMO‐1 at E318, K182, and K316 residues.^[^ ^26^, ^43^ ^]^ This process is also catalyzed by PIAS3 and has been observed in melanoma and renal cell carcinoma.^[^ ^26^, ^44^, ^45^ ^]^ Unlike SUMO3, the SUMO‐1‐related sumoylation of MITF does not influence its ubiquitination^[^ ^26^ ^]^ but does inhibit its transcriptional activity.^[^ ^44^, ^45^ ^]^ Mutations at E318, K182, and K316 sites that block SUMO‐1‐related sumoylation lead to increased oncogenic activity of MITF and are associated with a higher risk of melanoma and renal cell carcinoma.^[^ ^43^ ^]^ The lack of SUMO‐1 in promoting ubiquitination may be due to its inability to form polymers, which can be attributed to the absence of a classic sumoylation consensus motif (ψKXE, where ψ represents aliphatic amino acids and X can be any amino acid) when compared to SUMO3.^[^ ^25^, ^46^ ^]^ In addition to sumoylation, MITF can be O‐GlcNAcylated at the S49 residue in breast cancer, which promotes palbociclib‐induced senescence and contributes to therapy resistance.^[^ ^47^ ^]^ In esophageal squamous cell carcinoma (ESCC), MITF has been observed to be phosphorylated at Y360 by CLK4. This phosphorylation enables SQSTM1/p62‐mediated recognition and autophagic degradation of MITF, thereby inhibiting the progression of ESCC.^[^ ^5^ ^]^ Furthermore, MITF can be acetylated by the p300/CREB‐binding protein, which supports the development of melanocytes and drives tumorigenesis.^[^ ^48^ ^]^ These modifications, along with the sumoylation of MITF by SUMO3, highlight a critical and distinct regulatory mechanism in controlling MITF stability and function across various cancer types.

Our findings indicate that high levels of METTL10 expression may serve as a diagnostic marker for advanced, progressive forms of GC, particularly in refractory GC (RGC), which shows poor responses to standard treatments and experiences frequent relapses within short timeframes. In mouse models of advanced GC, we identified the METTL10‐PIAS3‐MITF axis as a key driver of GC malignancy. Importantly, the compound, LQZ‐02‐023‐01 has shown promising therapeutic effects in both advanced GC (*Cldn18‐AT*) and inflammation‐induced GC (*K19‐C2mE*) mice. This suggests its potential as a treatment option for clinically RGC, although its specific clinical efficacy requires further investigation.

## 4 Experimental Section

### Mice Model

All experimental procedures were conducted in strict accordance with the guidelines established by the Animal Ethics Committee of Xiamen University. METTL10^−/−^ mice were generated and provided by the Xiamen University Laboratory Animal Center. Briefly, two single guide RNAs (sgRNAs) targeting exons 2 and 3 of the METTL10 gene (METTL10‐sgRNA‐F: gtataggagtgttcatccgatgg; and METTL10‐sgRNA‐R: tacgtgggtagcccctatggagg) were designed, leading to the deletion of exons 2 and 3 along with excision of an approximately 2650‐bp genomic fragment. The sequences of primers for mouse genotyping used are shown in Table S4 (Supporting Information). The *K19‐C2mE* was a gift from Prof. Masanobu Oshima. *Cldn18* ^CreERT2^; *Apc* ^fl/fl^; *Trp53* ^fl/fl^; *Kras* ^G12D^ (*Cldn18‐ATK*) mice were a gift from Dr. Yoshiaki Ito. To generate spontaneous GC models deficient in METTL10, METTL10^−/−^ mice were crossed with *K19‐C2mE* mice. The resulting METTL10^−/−^; *K19‐C2mE* mice developed spontaneous gastric tumors. The sequences of primers for mouse genotyping used are shown in Table S4 (Supporting Information). 6 to 8 week‐old female BALB/c nude mice were purchased from Beijing Vital River Laboratory Animal Technologies. All mice were bred under specific pathogen‐free (SPF) conditions at the Xiamen University Laboratory Animal Center. They were born and maintained within the same controlled environment and facilities characterized by a constant temperature of 22 °C and a 12‐h light/12‐h dark cycle, with unrestricted access to standard rodent chow and drinking water.

### Cell Culture

GES‐1 cells, HEK293T, MKN45, AGS, HGC‐27, MKN7, and MKN74 were obtained from Procell Life Science and Technology, China. GES‐1, MKN45, AGS, HGC‐27, MKN7, and MKN74 were cultured in RPMI‐1640 medium (Pricella) supplemented with 10% (v/v) fetal bovine serum (Viva Cell) and 1% (v/v) penicillin‐streptomycin solution (Pricella). HEK293T cells were maintained in DMEM (Pricella) supplemented with 10% (v/v) fetal bovine serum (Biological Industries) and 1% penicillin‐streptomycin solution (Pricella). All cells were incubated at 37 °C in 5% CO_2_.

### Plasmids Construction and Transfection

All plasmids were constructed by cloning the respective gene sequences into their corresponding vectors using molecular cloning techniques. Lentiviral vectors encoding short hairpin RNAs (shRNAs) targeting METTL10 and control vectors were synthesized by Unibio Corporation. Similarly, lentiviral vectors encoding shRNAs targeting MITF and control vectors were obtained from the Public Protein/Plasmid Library. The shRNA‐expressing plasmids targeting METTL10 or MITF were transfected into GC cell lines AGS and MKN45. Stable cell lines with knockdown of METTL10 or MITF were selected using 1 µg/ml puromycin over 2 weeks, and knockdown efficiency was verified by Western blot analysis. All mutant constructs were generated by ligation‐independent cloning (LIC) using the pcDNA3.1(+)‐Flag‐tag vector as the backbone. The plasmids were transfected into HEK293T cells or GC cells using ExFect Transfection Reagent (Vazyme, T101) according to the manufacturer's instructions. All the information on primers and plasmids is shown in Tables S4 and S5 (Supporting Information).

### Cell Proliferation Assay

Cell proliferation was assessed using Cell Counting Kit‐8 (CCK‐8; K1018, APExBIO). Briefly, 1000 cells per well were seeded into 96‐well plates, and proliferation was measured at 450 nm on days 0, 1, 3, 5, and 7 using the CCK‐8 assay using a multifunctional microplate reader.

### Cell Colonization Assay

Cells (1000 cells/well) were seeded in six‐well plates and provided with fresh culture medium every 3 days. After 14 days of incubation, the medium was removed, and the cells were gently washed three times with room‐temperature PBS. The cells were then fixed with 1 mL of 4% paraformaldehyde per well for 15 min. Next, 1 mL of 0.1% crystal violet solution was added to each well and incubated for 10 min to stain the cells. The crystal violet solution was drained, and the wells were rinsed with running water to remove excess stain, followed by air drying and photography.

### Wound Healing Assay

MKN45 and AGS cells were seeded into six‐well plates and cultured under standard conditions until a confluent monolayer was achieved. A linear wound was created by scraping the cell monolayer with a sterile 200‐µL pipette tip. Detached cells were removed by washing the wells three times with prewarmed phosphate‐buffered saline (PBS). Subsequently, 2 mL of serum‐free RPMI‐1640 medium was added to each well to minimize proliferation effects. Wound closure was monitored by capturing images at 0, 6, 12, 18, and 24 h using an inverted microscope. The wound width at each time point was quantified using ImageJ software, and migration rates were calculated accordingly.

### Transwell Migration and Invasion Assays

Cell migration and invasion assays were performed using 24‐well Transwell chambers (8‐µm pore size, Corning). For the migration assay, 700 µL of RPMI‐1640 medium supplemented with 10% FBS was added to the lower chamber as a chemoattractant. Then, 1 × 10⁴ cells suspended in 200 µL of serum‐free RPMI‐1640 medium were seeded into the upper chamber. After incubation for 48 h at 37 °C, nonmigrated cells remaining on the upper surface of the membrane were gently removed with a cotton swab. Cells that had migrated to the lower surface were fixed with 4% paraformaldehyde, stained with 0.1% crystal violet, and imaged under a microscope for quantification. For the invasion assay, the upper surface of the membrane was precoated with 50 µL of Matrigel (Corning), diluted in serum‐free medium according to the manufacturer's instructions. The remaining procedures were identical to those used in the migration assay. The number of migrated or invaded cells was quantified using ImageJ software.

### RNA Extraction, Reverse‐Transcription, and RT‐qPCR

According to the instructions of the manufacturer, total RNA was extracted from tissues or cells with RNA pure Tissue and Cell Kit (CWBIO, CW0584S). The 2 µg RNA was then reverse‐transcribed into cDNA using HiFi‐MMLV cDNA Kit (CWBIO, CW0744M). RT‐qPCR was performed by UltraSYBR Mixture (CWBIO, CW0957H) using Bio‐Rad C1000 Thermal Cycler CFX96. The sequences of primers for the target gene used are shown in Table S4 (Supporting Information).

### RNA Sequencing

RNA sequencing was performed by GENE DENOVO Biotechnology (Guangzhou, China). Total RNA was extracted from METTL10‐overexpressing MKN45 cells. Following quality control using an Agilent 2100, 5 µg of RNA was employed to construct paired‐end (PE) libraries with the Ultima Dual‐mode mRNA Library Prep Kit (Yeasen). The quality of the libraries was evaluated with a DNA 1000 Assay Kit (Agilent Technologies). Sequencing was conducted on the Illumina NovaSeq 6000 platform, and the resulting data were subsequently analyzed using the Omicsmart platform for bioinformatics evaluation. The RNA sequencing data produced in this study have been deposited in the National Genomics Data Center (NGDC) under the accession number: PRJCA030347.

### Immunoblotting and Immunoprecipitation

Protein extraction from tissues or cells was performed by lysis buffer (20 mM Tris‐HCl (pH 7.5), 150 mM NaCl, 1 mM EDTA, 1 mM EGTA, 2.5 mM sodium pyrophosphate, 1 mM β‐glycerolphosphate, 1% Triton X‐100) supplemented with 1% phenylmethylsulfonyl fluoride (PMSF; Solarbio, P0100) and protease inhibitor cocktail (Apexbio, K1007) and lysed at 4 °C for 60 min, followed by centrifugation at 12000 g for 15 min at 4 °C. For Immunoblotting, the protein concentration was determined using the Pierce BCA Protein Assay Kit (Thermo Fisher Scientific, A55864). The protein samples were denatured by boiling in SDS‐PAGE loading buffer. For immunoprecipitation, antibodies were incubated with Dynabeads Protein G (Thermo, 10004D) at 4 °C for 4 h. The supernatant was then incubated with magnetic beads conjugated with antibodies or anti‐FLAG‐M2 magnetic beads (Sigma–Aldrich, M8823) at 4 °C overnight. Following incubation, the magnetic beads were washed three times with lysis buffer and then boiled in SDS‐PAGE loading buffer. The proteins were separated by sodium dodecyl sulfate polyacrylamide gel electrophoresis (SDS‐PAGE) with One‐Step PAGE Gel Fast Preparation Kit (Vazyme), and then transferred to the PVDF Membrane (Bio‐Rad). After blocking with a protein‐free rapid blocking buffer (Epizyme, PS108P) for 15 min, the membranes were incubated with primary antibodies at 4 °C overnight. The membranes were washed with Tris‐Buffered‐Saline with Tween (TBST) three times, and then incubated with secondary antibodies at room temperature for 2 h. The immunoblot bands were visualized using an electrochemiluminescence (ECL) assay kit (Bio‐Rad Clarity Western ECL Substrate). Raw images were acquired with the Azure C280, Azure C300, or GS‐600UV chemiluminescent imaging systems, using Azure Biosystems or GelView software during automated capture. Detailed antibody and reagent information is shown in Table S6 (Supporting Information).

### Protein Half‐Life Assay

MKN45 and AGS cells overexpressing Flag‐METTL10 or Flag‐Vector were treated with cycloheximide (CHX) at a final concentration of 12.5 µM for 0, 6, 12, 24, and 36 h. Immunoblotting was employed to assess the levels of MITF, which were subsequently normalized to β‐actin using ImageJ software.

### In Vivo Ubiquitination

Plasmids were transfected into HEK293T cells for 24 h or into gastric cells for 36 h. Following transfection, cells were treated with 10 µM MG132 (Selleck, S2619) for 12 h to inhibit proteasome‐mediated degradation before harvesting. After lysing the cells with lysis buffer, the samples were subjected to immunoprecipitation and analyzed by immunoblotting.

### Protein Purified

The GST‐tagged and His‐tagged proteins were expressed in BL21 bacterial cells (Sangon) and purified using Glutathione Agarose Resin (Thermo Fisher) or Ni‐NTA Resin (Thermo Fisher), respectively. Flag‐tagged MITF was expressed in HEK293T cells and lysed in a buffer containing 20 mM Tris‐HCl (pH 7.5), 150 mM NaCl, 1 mM EDTA, 1 mM EGTA, 2.5 mM sodium pyrophosphate, 1 mM β‐glycerolphosphate, and 1% Triton X‐100, supplemented with 1% PMSF and a protease inhibitor cocktail. The protein was purified by affinity chromatography using anti‐FLAG‐M2 magnetic beads, followed by washing three times with lysis buffer and elution with 3x FLAG peptides (MCE).

### GST Pulldown

Purified GST or GST‐PIAS3 proteins were incubated with purified His‐METTL10 in lysis buffer (25 mM Tris‐HCl (pH 8.0) and 150 mM NaCl) at 4 °C overnight. This mixture was then incubated with Glutathione Agarose Resin at 4 °C for 4 h. The resin was washed three times with lysis buffer, and GST‐tag proteins were eluted with elution buffer (25 mM Tris‐HCl (pH 8.0), 150 mM NaCl, and 10 mM Reduced Glutathione). The eluates were subsequently boiled in SDS‐PAGE loading buffer for immunoblotting.

### In Vitro Sumoylation and Ubiquitination

For the in vitro Sumoylation assay, bacteria‐purified GST‐PIAS3 and HEK293T‐purified MITF were incubated with SAE1, UBC9 in reaction buffer according to the manufacturer's instructions at 37 °C for 2 h. For the in vitro ubiquitination assay, purified His‐METTL10, GST‐PIAS3, Flag‐MITF, SAE1, and UBC9 proteins were incubated with ubiquitin, UBE1, UBE2B, and RNF4 in reaction buffer according to the manufacturer's instructions at 37 °C for 2 h. All the samples were subjected to immunoprecipitation and subsequently boiled in SDS‐PAGE loading buffer for immunoblotting.

### In Vitro Methylation

For the in vitro methylation assay, His‐METTL10 (1 µg) was incubated with recombinant human Histone H4 protein (20 ng; Abcam, ab198115) or GST‐PIAS3 (100 ng) of in methylation reaction buffer (50 mM Tris‐HCl (pH 8.0), 20 mM KCl, 5 mM DTT, 4 mM EDTA) containing 20 µM S‐(5′‐adenosyl)‐L‐methionine chloride dihydrochloride (SAM) at 37 °C for 1 h. The reaction was terminated by boiling the samples in SDS‐PAGE loading buffer, and the products were analyzed by immunoblotting.

### Interaction Protein and Methylation Sites Detection by LC‐MS/MS

To detect interacting proteins, MKN45 cells were transfected with either Flag‐METTL10 or Flag‐Vector plasmids for 36 h. For methylation site detection, HEK293T cells were co‐transfected with Flag‐METTL10 and HA‐PIAS3 plasmids or Flag‐Vector and HA‐PIAS3 plasmids for 24 h. The cells were lysed with lysis buffer containing 1% PMSF and protease inhibitor cocktail at 4 °C for 60 min, followed by centrifugation at 12 000 g for 15 min at 4 °C. The resulting supernatant was incubated with Flag‐M2 magnetic beads at 4 °C overnight. After incubation, the beads were washed three times with lysis buffer and subsequently boiled in SDS‐PAGE loading buffer. The proteins were separated by SDS‐PAGE and visualized using Coomassie blue staining. For interacting protein detection, the entire gel lanes were excised and analyzed by LC‐MS/MS. For methylation site detection, the PIAS3 protein bands were excised and subjected to LC‐MS/MS. The identification of interacting proteins and methylation sites with METTL10 was performed using a Bruker timsTOF Pro ion mobility mass spectrometer for LC‐MS/MS analysis. The proteomics data produced in this study have been deposited in the ProteomeXchange Consortium via the PRIDE partner repository under the accession number: PXD057247.

### Analysis of Metabolites by LC‐MS

Cells were collected and transferred to centrifuge tubes, followed by three washes with cold PBS. The samples were then resuspended in an extraction solution (methanol: acetonitrile: water = 2:2:1, v/v/v, 1 mL) and vortexed for 30 s. The suspension underwent ice bath sonication for 10 min and frozen and thawed in liquid nitrogen for repeated three times. To fully precipitate proteins, the samples were incubated at −20 °C for 1 h. Subsequently, the cell lysates were centrifuged at 13000 rpm for 15 min at 4 °C. The supernatant was then lyophilized using a low‐temperature vacuum centrifugal concentrator and analyzed by LC‐MS using the AB SCIEX QTRAP 6500^+^ system.

### Animal Studies

METTL10^WT^ or METTL10^−/−^ *K19‐C2mE* spontaneous GC mice were generated and scarified at 30 weeks. Subcutaneous tumor models were established by subcutaneous injection of transfected MKN45 (5 × 10^6^/100 µL) and AGS (1 × 10^7^/100 µL) cells into the flank of BALB/c nude mice. For MITF rescue subcutaneous tumor models, transfected MKN45 (5 × 10^6^/100 µL) cells were injected subcutaneously into the flank of BALB/c nude mice.

For PIAS3‐K442 rescue subcutaneous tumor models, transfected MKN45 (5 × 10^6^/100 µL) cells were injected subcutaneously into the flank of BALB/c nude mice. All mice were monitored every 2 days and sacrificed after 18 days.

For the subcutaneous tumor model, 5 × 10⁶ MKN45 cells suspended in 100 µL of sterile PBS were injected subcutaneously into the flanks of BALB/c nude mice. When tumor volumes reached approximately 50 mm^3^, mice were randomized into three groups (Vehicle control, LZQ‐02‐023‐01 at 20 mg/kg or 40 mg/kg, *n* = 10 per group). All treatments were administered via intraperitoneal injection once daily for 14 consecutive days. Tumor volumes were measured every 2 days using calipers and calculated using the formula: volume = [length × (width^2^)]/2. Mice were sacrificed on day 24 for endpoint analyses. For genetically engineered mouse models (GEMMs), 25‐week‐old *K19‐C2mE* mice were randomized into Vehicle control (*n* = 8) and LZQ‐02‐023‐01 treatment (40 mg/kg, *n* = 8) groups. Both groups received daily intraperitoneal injections for 28 consecutive days. Similarly, 8‐week‐old *Cldn18‐AT* mice were induced with tamoxifen (100 mg/kg, T5648, Sigma) and, 2 weeks post‐induction, randomized into Vehicle (*n* = 6) and LZQ‐02‐023‐01 (40 mg/kg, *n* = 6) treatment groups. Treatments were administered daily via intraperitoneal injection for 28 days.

For the establishment of lung metastasis, 5×10⁶ MKN45 cells were resuspended in 100 µL of PBS and administered via lateral tail vein injection into mice. After 30 days, mice were euthanized, and lungs were perfused with PBS, harvested, and subjected to gross morphological examination and hematoxylin and eosin (H&E) staining. Metastatic lesions were identified and confirmed by both macroscopic inspection and histopathological analysis. Quantification of metastatic nodules and metastatic area in lung sections was performed using ImageJ software.

Orthotopic gastric cancer models were established by surgically implanting 1 × 10⁶ METTL10‐overexpressing or vector control ATK cells into the gastric subserosal layer of C57BL/6 mice under aseptic conditions. Tumor tissues were harvested 20 days post‐implantation for subsequent analyses. In a separate cohort, mice bearing orthotopic tumors (1 × 10⁶ cells implanted) received subcutaneous administration of LZQ‐02‐023‐01 at a dose of 40 mg/kg/day, starting 7 days after tumor implantation. The treatment continued daily for 14 consecutive days. Tumor burden and therapeutic response were assessed at the end of the treatment period by excision and analysis of gastric tumors.

For the acute toxic study, 8‐week‐old ICR mice (*n* = 6 per group) were administered a single intraperitoneal injection of LZQ‐02‐023‐01 at escalating doses. A vehicle‐treated group served as control. Survival was monitored continuously for 24 h postadministration. Additionally, another cohort of ICR mice (*n* = 5 per group) received a single intraperitoneal dose of LZQ‐02‐023‐01 at 125 mg/kg and were subsequently monitored daily for 7 days for clinical signs of toxicity, behavioral changes, body weight fluctuations, and mortality. At the conclusion of the observation period, animals were euthanized and subjected to a comprehensive gross necropsy. Major organs, including the liver, kidney, heart, lung, and spleen, were harvested for histopathological examination. For the chronic toxic study, 8‐week‐old ICR mice (*n* = 5 per group) received daily intraperitoneal injections of LZQ‐02‐023‐01 at a therapeutic dose of 40 mg/kg for 14 consecutive days. A vehicle‐treated group was included as a control. Throughout the treatment period, animals were monitored daily for clinical signs of toxicity and weighed every 2 days. At study termination, blood samples were collected for hematological and biochemical analyses. A comprehensive necropsy was performed, and major organs were harvested for detailed histopathological evaluation to identify any potential toxicological effects associated with repeated dosing. All animal procedures were reviewed and approved by the Laboratory Animal Center of Xiamen University (Approval No. XMULAC20200181), and were carried out in accordance with the National Guidelines for Animal Care and Use.

### Flow Cytometry Analysis of Tumor‐Infiltrating Cells

Tumor tissues were finely minced into approximately 1 mm^3^ pieces and enzymatically digested in Hank's Balanced Salt Solution (HBSS) containing type IV collagenase (2357210, Gibco) at 37 °C for 1 h. The resulting cell suspensions were gently triturated and passed through an 80 µm cell strainer (22131209, Biosharp) to remove debris. Red blood cells were lysed using ACK lysis buffer (R1010, Solarbio) to yield a single‐cell suspension. Cells were subsequently stained with a cocktail of fluorochrome‐conjugated antibodies (1:200 dilution; details listed in Table S6, Supporting Information) and a fixable viability dye (1:200, 2365395, Invitrogen) for 30 min at 4 °C in the dark. After washing, the stained cells were analyzed using a Sony ID7000 spectral flow cytometer (Sony Biotechnology).

### HE Staining and IHC Staining

Paraffin sections were initially placed in an oven at 65 °C for 30 min to melt the paraffin. The slides were then immersed in two consecutive xylene baths for 10 min each to dewax the tissue, followed by hydration through a series of graded ethanol solutions. The sections were stained with hematoxylin for 10 min. After differentiation with 1% hydrochloric acid alcohol, the sections were blued with lithium carbonate and counterstained with eosin for 3 min. The slides were then dehydrated in graded ethanol and mounted with neutral resin. All pathological results were performed in a blinded manner by experienced gastrointestinal pathologists. For IHC staining, sections were processed in accordance with the manufacturer's protocol (MXB Biotechnologies). Briefly, antigen retrieval was conducted at 37 °C for 30 min, followed by blocking at room temperature for 10 min. The sections were subsequently incubated with the appropriate primary antibody overnight at 4 °C. The following day, biotin‐conjugated secondary antibodies were applied and incubated for 1 h, followed by streptavidin‐peroxidase incubation for 10 min. Diaminobenzidine (DAB) was employed as the chromogen for visualization, and hematoxylin was used for counterstaining. Imaging was performed using the Olympus VS200 microscope.

### Methylated RNA Immunoprecipitation (MeRIP)‐qPCR

The MeRIP‐qPCR assay was performed using the riboMeRIP m^6^A Transcriptome Profiling Kit (RIBOBIO, R11096.6). In brief, total RNA was extracted and fragmented, with a portion set aside as input. Magnetic A/G beads were incubated with an m^6^A‐specific antibody, followed by washing and subsequent incubation with the RNA sample at 4 °C for 2 h. After thorough washing, the RNA was eluted from the beads and subjected to RT‐qPCR analysis. The potential m^6^A modification sites on PIAS3 were predicted using the SRAMP tool (http://www.cuilab.cn/sramp/). The sequences of primers for the target gene used are shown in Table S4 (Supporting Information).

### Luciferase Reporter Assay

The potential MITF binding sites on the METTL10 promoter were predicted using LASAGNA‐Search 2.0 (https://biogrid‐lasagna.engr.uconn.edu/lasagna_search/). The top two sites with the highest confidence scores were individually or simultaneously mutated. Luciferase reporter plasmids integrating firefly and Renilla luciferase genes were constructed by Unibio Corporation: METTL10 promoter WT pDualuc‐Basic, METTL10 promoter MUT1 pDualuc‐Basic, METTL10 promoter MUT2 pDualuc‐Basic, and METTL10 promoter DM pDualuc‐Basic. These plasmids were transfected into HEK293T, AGS, or MKN45 cells and incubated for 36 h prior to performing the luciferase assay. Luciferase activity was assessed using the Dual‐Luciferase Reporter Assay System (Promega, E1910), with firefly luciferase activity normalized to Renilla luciferase activity. All experiments were conducted in triplicate.

### Chromatin Immunoprecipitation (ChIP)‐qPCR

A ChIP assay was performed using anti‐MITF antibody (Abcam, ab12039), and the operating process was performed according to the manufacturer's instructions for the Pierce Magnetic ChIP Kit (Thermo Fisher Scientific). Fold enrichment was quantified by RT‐qPCR and calculated as a percentage of input chromatin. The sequences of primers for the target gene used are shown in Table S4 (Supporting Information).

### Screening iMETTL10 Based on MTase‐Glo Assay

To identify METTL10 inhibitors, an in‐silico screening was first conducted on an in‐house compound library comprising approximately 6000 structurally diverse molecules. Molecular docking simulations with the METTL10 protein identified 111 candidate compounds for further analysis. The methyltransferase activity of these candidates was assessed using the MTase‐Glo Methyltransferase Assay (Promega, V7601) according to the manufacturer's protocol. Briefly, the candidate compounds were preincubated with His‐METTL10 protein (1 µg) at 37 °C for 1 h in a methylation reaction buffer (50 mM Tris‐HCl (pH 8.0), 20 mM KCl, 5 mM DTT, 4 mM EDTA, and 20 µM SAM), followed by the addition of either the Histone H4 or PIAS3 peptide substrate for an additional 1 h. The reaction was then subjected to MTase‐Glo reagent buffer for 30 min, followed by MTase‐Glo detection buffer for another 30 min. Luminescence was measured using a luminometer (Promega), and IC_50_ values were calculated using GraphPad Prism 8 software.

### Chemical Synthesis of iMETTL10

Three‐(3‐aminophenyl)‐1‐methyl‐7‐((6‐methylpyridin‐3‐yl) amino)‐3,4‐dihydropyrimido [4,5‐d] pyrimidin‐2 (1H)‐one (LZQ‐02‐023‐01). A mixture of IR‐2 (1 g, 3.46 mmol), Di‐tert‐butyl decarbonate (1.51 g, 6.92 mmol), 4‐Dimethylaminopyridine (42 mg, 0.346 mmol and triethylamine (700 mg, 6.92 mmol) in DMSO (Dimethyl sulfoxide, 40 ml) was heated at 60 °C for 4 h. After the reaction was complete asmonitored by LC‐MS, the resulting mixture was filtered by H_2_O through a celite pad, and the filtrate was concentrated to give the crude product. The crude product was purified by silica‐gel column chromatography (DCM:MeOH = 86:14) to give Compound 1 (1.1 g, 2.83 mmol, 81.7%). To a mixture of compound 1 (1.1 g, 2.83 mmol), 6‐methylpyridin‐3‐amine (220 mg, 2 mmol), Pd_2_ (DBA)_3_ (466 mg, 0.5 mmol), X‐Phos (365 mg, 0.76 mmol) and K_2_CO_3_ in t‐BuOH (tert‐Butanol, 10 mL) under N_2_ was heated at 110 °C in a seal tube for 10 h. After the reaction was complete, as monitored by LC‐MS, the resulting mixture was filtered through a celite pad, and the filtrate was concentrated to give the crude product. The crude product was purified by silica‐gel column chromatography (DCM:MeOH = 90:10) to give Compound 2 (0.8 g, 1.73 mmol, 86.7%).

A round‐bottom flask was charged with compound 2 (0.8 g, 1.73 mmol), Trifluoroacetic acid (2 mL) in DCM (10 mL) at 25 °C. After 3 h, LC‐MS showed a new peak with the desired mass ([M + H] = 362.1) was detected. The residue was partitioned between DCM (100 mL) and NaHCO_3_ (aq., 20 mL). The organic layer was washed with water, dried (Na_2_SO_4_), and evaporated to dryness. The residue was purified by silica‐gel column chromatography to give the Compound LZQ‐02‐023‐01 (450 mg, 1.25 mmol, 72.0%).

^1^H NMR (600 MHz, DMSO‐d_6_) δ 2.40 (s, 3H), 3.31 (s, 3H), 4.63 (s, 2H), 5.16 (s, 2H), 6.45–6.49 (m, 2H), 6.53 (t, *J* = 2.1 Hz, 1H), 7.03 (t, *J* = 7.9 Hz, 1H), 7.17 (d, *J* = 8.4 Hz, 1H), 8.04 (dd, *J* = 8.4, 2.7 Hz, 1H), 8.14 (s, 1H), 8.77 (d, *J* = 2.7 Hz, 1H), 9.59 (s, 1H). ^13^C NMR (150 MHz, DMSO‐d_6_) δ 158.96, 156.91, 153.11, 152.73, 150.21, 149.26, 143.31, 140.02, 134.77, 129.12, 126.28, 122.53, 112.68, 112.02, 111.28, 103.31, 46.52, 28.32, 23.24. HRMS (m/z): [M + H]^+^ calculated for C_19_H_19_N_7_O^+^, 362.1685; found, 362.1717.

### Molecular Docking

The 3D structure of METTL10 was retrieved from the AlphaFold Protein Structure Database (https://alphafold.ebi.ac.uk/). Molecular docking simulations were conducted using the Maestro 11.1 software suite (Schrödinger, LLC). In the docking process, METTL10 was treated as a rigid receptor, while the ligand molecule LZQ‐02‐023‐01 was modeled with full conformational flexibility. Binding affinities and interaction profiles between METTL10 and LZQ‐02‐023‐01 were assessed. The resulting docking poses were further analyzed using LigPlot (v2.2), which generated 2D interaction diagrams highlighting key hydrogen bonds and hydrophobic contacts between the ligand and the surrounding amino acid residues.

### Microscale Thermophoresis (MST)

Microscale thermophoresis (MST) assays were conducted following the protocol.^[^ ^49^ ^]^ Briefly, HEK293T cells were transfected with either the pEGFP‐N1‐METTL10 plasmid or the control pEGFP‐N1‐Vector plasmid for 24 h. Subsequently, protein lysates were obtained through ultrasonic disruption at 4 °C. Aliquots of the lysate were then incubated with serially diluted ligands at 37 °C for 30 min. Fluorescence signals were measured, and dissociation constants (Kd) were determined using the NanoTemper Monolith Pico.

### Off‐Target Methyltransferase Profiling of LZQ‐02‐023‐01

To evaluate the off‐target effects of LZQ‐02‐023‐01 on methyltransferase activity, a panel of enzymes was assessed using the MTase‐Glo Methyltransferase Assay (Promega), following the manufacturer's instructions. The assay was performed by ICE Bioscience Inc. Briefly, LZQ‐02‐023‐01 was serially diluted 4‐fold in DMSO to generate a 10‐point concentration gradient, and the resulting solutions were dispensed into 384‐well white assay plates using an Echo acoustic liquid handler (Labcyte). Plates were centrifuged at 1000 rpm for 1 min to ensure even compound distribution. Following a 10‐min preincubation at 25 °C, 2 µL of a substrate solution containing S‐adenosylmethionine (SAM) and the specific methyltransferase substrate (Table S7, Supporting Information) was added to each well. After a brief centrifugation, plates were incubated at 25 °C for 180 min to allow the enzymatic methylation reaction to proceed. Subsequently, 1 µL of MTase‐Glo reagent was added, followed by centrifugation and incubation at 25 °C for 45 min. Then, 5 µL of MTase‐Glo detection solution was introduced into each well, and the plates were incubated for an additional 45 min at 25 °C after centrifugation.

Luminescence signals were directly proportional to methyltransferase activity, were recorded using a microplate reader (BMG LABTECH). Percent inhibition was calculated by normalizing luminescence intensities of compound‐treated wells to DMSO‐treated controls, and IC_50_ values were calculated using GraphPad Prism 8 software.

### Pharmacokinetics of LZQ‐02‐023‐1

LZQ‐02‐023‐1 was administered to male Sprague–Dawley (SD) rats (*n* = 3 per group) via intravenous injection at a dose of 1 mg/kg or oral gavage at a dose of 10 mg/kg. Blood samples were collected at predetermined time points, and plasma concentrations of LZQ‐02‐023‐1 were determined using a validated LC‐MS/MS method. Briefly, working solutions of the analyte were prepared by diluting stock solutions with 50% acetonitrile in water to achieve serial concentrations (10, 20, 50, 100, 200, 500, 1000, 5000, and 10 000 ng/mL). Calibration standards were prepared by spiking 5 µL of working solution into 50 µL of blank SD rat plasma, resulting in final concentrations of 1, 2, 5, 10, 20, 50, 100, 500, and 1000 ng/mL in a total volume of 55 µL. Quality control (QC) samples were independently prepared at four concentrations (2, 5, 50, and 800 ng/mL) using blank SD rat plasma, following the same procedure as for the calibration standards.

On the day of analysis, 55 µL of each calibration standard, QC sample, or unknown sample (prepared by mixing 50 µL of SD rat plasma with 5 µL of blank solution) was added to 200 µL of acetonitrile:methanol (1:1, v/v) containing an internal standard (IS) mixture for protein precipitation. The samples were vortexed for 30 s, centrifuged at 4000 rpm for 15 min at 4 °C, and the resulting supernatant was diluted threefold with water. A 10 µL aliquot of the diluted supernatant was injected into an AB Sciex Triple Quad 5500 LC‐MS/MS system for quantitative analysis. Pharmacokinetic parameters, including the maximum plasma concentration (Cmax), time to reach Cmax (Tmax), elimination half‐life (t_1_⁄_2_), area under the plasma concentration‐time curve (AUC_0_–t and AUC_0_–∞), clearance (CL), and volume of distribution (Vd), were calculated using noncompartmental analysis (NCA) in Phoenix WinNonlin 8.3 (Certara; Table S3, Supporting Information).

### Generation of PIAS3^K442‐Methyl^ Specific Antibody

The rabbit monoclonal antibody against PIAS3^K442‐Methyl^ was customized and developed by Nanjing GenScript Technologies Co., Ltd, against the synthetic peptide CVQGGDPSENK(Me)KKVE, where the lysine 442 residue was methylated. Briefly, a peptide that encompasses the amino acids surrounding the K442 site of PIAS3, specifically methylating lysine 442 (CVQGGDPSENK(Me)KKVE), was synthesized. Rabbits were immunized with this methylated peptide following a standard immunization protocol over 10 weeks. The resulting antisera were then affinity‐purified using the same methylated peptide immobilized on a column to isolate the methylation‐specific antibodies. Western blot analysis confirmed that the antibody specifically detects PIAS3 only when lysine 442 was methylated (PIAS3^K442‐Methyl^; Figure 4d,e; Figure S4h, Supporting Information).

### Clinical Samples

Paired tissue samples of GC patients undergoing surgery from Zhongshan Hospital of Xiamen University, relevant information on the patients is provided in Table S8 (Supporting Information). None of the patients underwent any prior therapeutic interventions for GC prior to undergoing surgery. The resected tissues were immediately preserved by freezing in liquid nitrogen or fixing in 4% paraformaldehyde, and subsequently subjected to thorough histological and pathological assessment and grading by an experienced pathologist. This study received approval from the Ethics Committee of the Zhongshan Hospital of Xiamen University (Approval No. xmzsyyky‐2022‐160), and all tissue samples were collected with informed consent from the participants.

### Bioinformatics Analysis

Transcriptomic and clinical data were retrieved from publicly available datasets, including The Cancer Genome Atlas (TCGA) and Gene Expression Omnibus, including GSE118916, GSE65801, and GSE13195. Differential expression analysis of the seven β‐set (7BS) methyltransferase family genes was performed using the “limma” package within the R statistical environment (version 4.1.3). Visualization of gene expression patterns was achieved using the “ggplot2” package, and volcano plots were generated to illustrate the distribution of DEGs. Kaplan–Meier survival analyses for METTL10 and MITF were conducted using the KM plotter platform (http://kmplot.com/analysis/)

### Statistical Analysis and Reproducibility

Quantitative data are presented as mean ± standard deviation (S.D.). Statistical analyses were performed using GraphPad Prism 8.0. For comparisons between two groups, unpaired, two‐tailed student's *t*‐tests were used. For comparisons among multiple groups, one‐way ANOVA followed by Tukey's post hoc test was applied. Spearman correlation analysis was conducted using the two‐tailed Spearman rank correlation test. Survival analysis was performed using the log‐rank test. All statistical tests were two‐tailed, and *p* < 0.05 was considered statistically significant. Details regarding replicates are provided in the figures.

## Conflict of Interest

The authors declare no conflict of interest.

## Author Contributions

J.T., H.Z., J.M. contributed equally to this work. X.H., and X.D. conceived the project, generated hypotheses, and designed the studies. J.T., H.Z., and J.M. were the key contributors in designing and conducting most of the experiments. C.Z., M.Z., G.P., S.Z., and J.Z. provided constructive suggestions and help for this project. J.T. performed mouse gavage and drug treatment and conducted bioinformatics analysis. X.H. and Y.Z. provided clinical samples from GC patients. M.X. and W.Y. collected all clinical samples and clinical characteristics. A.C., Y.X., Y.Y., and Z.L. conducted HE staining and histological grading, and S.L., B.Z., and C.Y. conducted molecular docking simulations and molecular dynamics (MD) simulations for the preliminary screening of the drug library. Y.T. performed the chemical synthesis of LZQ‐02‐023‐01. H.Z. and J.T. wrote the manuscript, and X.H. and X.D. reviewed and edited the manuscript.

## Supporting information



Supporting Information

Supplemental Table 1

Supplemental Table 2

Supplemental Table 3

Supplemental Table 4

Supplemental Table 5

Supplemental Table 6

Supplemental Table 7

Supplemental Table 8

## Data Availability

The data that support the findings of this study are available from the corresponding author upon reasonable request.
