# Supplementary material for: METTL10‐Mediated PIAS3 Methylation Links Purine Metabolism to Gastric Cancer Progression
Source: Adv Sci (Weinh). 2025 Oct 20;12(48):e07054. doi: 10.1002/advs.202507054 (PMC12752606; doi:10.1002/advs.202507054)
Supplement: Supplementary file 1 — Supporting Information [file ADVS-12-e07054-s001.pdf]

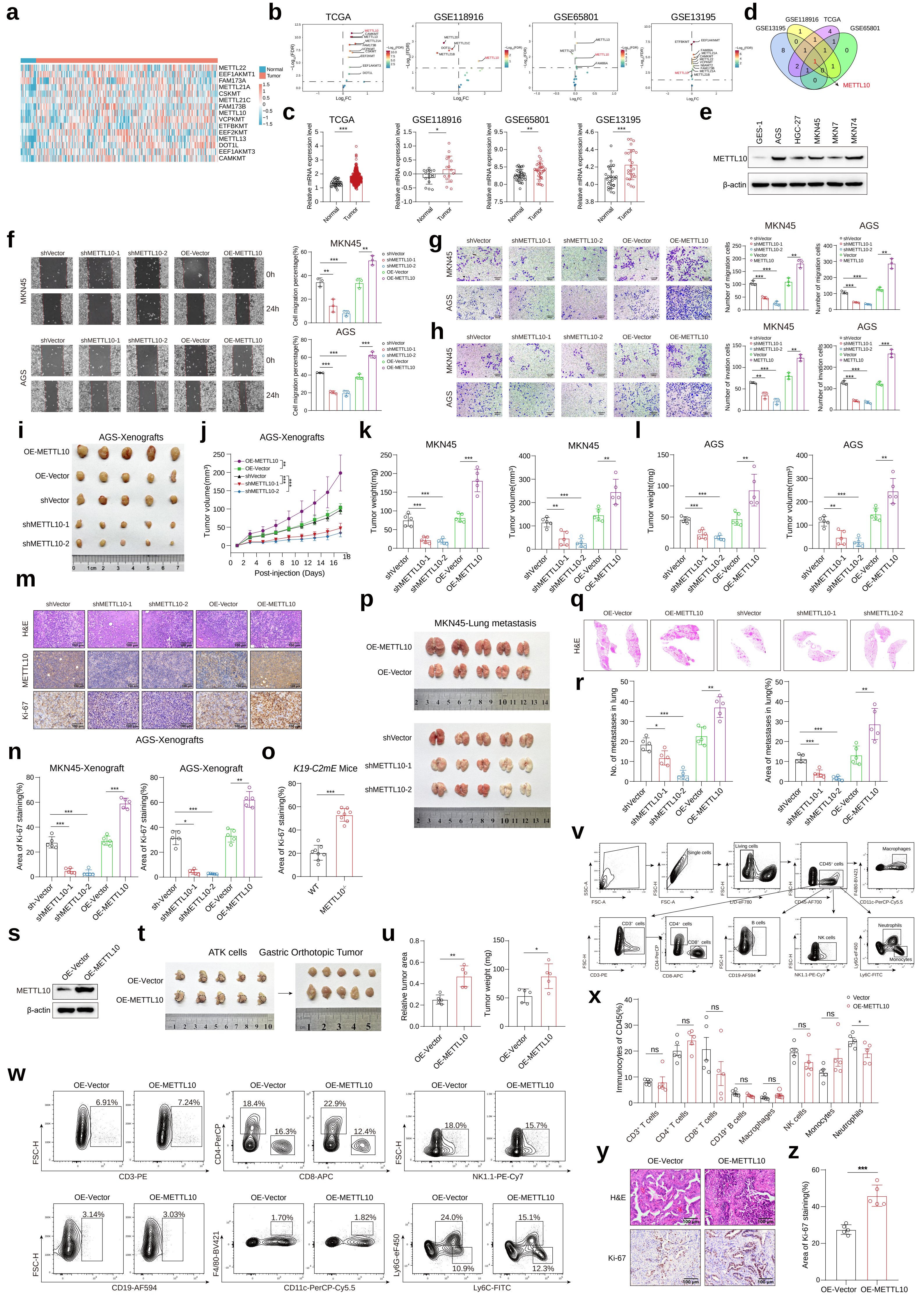

a
b
TCGA
GSE118916
GSE65801
GSE13195
c
d
e
METTL10
β-actin
GES-1
AGS
HGC-27
MKN45
MKN7
MKN74
f
shVector
shMETTL10-1
shMETTL10-2
OE-Vector
OE-METTL10
MKN45
AGS
0h
24h
g
h
i
AGS-Xenografts
j
k
l
m
H&E
Ki-67
n
MKN45-Xenograft
AGS-Xenograft
o
K19-C2mE Mice
p
MKN45-Lung metastasis
q
r
s
t
ATK cells
Gastric Orthotopic Tumor
u
v
w
x
y
z

Figure S1. METTL10 enhances malignancy in gastric cancer cells

a) Heatmap of human seven-β-strand methyltransferases in adjust normal (n= 32) and gastric cancer samples (n=375) based on TCGA.

b) Volcano plot of different seven-β-strand methyltransferases in TCGA, GSE118916, GSE65801, and GSE13195 datasets.

c) Comparative analysis of METTL10 mRNA expression in gastric cancer and adjacent normal tissues across TCGA, GSE13195, GSE65801, and GSE118916 datasets.

d) Venn diagram identifying METTL10 as the sole gene consistently upregulated in gastric cancer samples across TCGA, GSE13195, GSE65801, and GSE118916 datasets.

e) Western blot analysis of METTL10 protein expression in normal gastric epithelial cells (GES-1) and gastric cancer cell lines (AGS, HGC-27, MKN45, MKN7, and MKN74).

f) Wound-healing assay evaluating cell migration in MKN45 and AGS cells transfected with METTL10 or control vectors (n = 3, per group). Representative images were taken at 0 and 24 hours post-scratch.

g) Transwell migration assay of METTL10-transfected MKN45 and AGS cells (n = 3, per group).

h) Transwell invasion assay of METTL10-transfected MKN45 and AGS cells (n = 3, per group).

i) Photographs of xenograft tumors formed by subcutaneous injection of transfected AGS cells into Balb/c nude mice (n = 5, per group).

j) Tumor growth curves of AGS-derived xenografts measured over time (n = 5, per group).

k and **l**) Quantification of tumor volume and tumor weight in xenografts derived from MKN45 (**k**) and AGS (**l**) cells (n = 5, per group).

m) Representative histological and immunohistochemical images of xenograft tumors derived from AGS cells, stained with H&E, anti-METTL10, and Ki-67 antibodies. Scale bar, 100 μm.

n and **o**) Quantitative analysis of Ki-67-positive cells in xenograft tumors derived from MKN45 and AGS cells (**n**) and in tumors from WT or METTL10$^{-/-}$ *K19-C2mE* mice (**o**).

p) Photographs of pulmonary metastases following tail vein injection of METTL10-transfected MKN45 cells into BALB/c nude mice (n = 5, per group).

q) H&E staining of lung sections showing metastatic foci derived from MKN45-transfected cells.

r) Quantitative analysis of the number and area of lung metastatic nodules.

s) Western blot analysis confirming METTL10 overexpression in transfected ATK cells.

t) Photographs of gastric orthotopic allograft tumors induced by injecting METTL10-transfected ATK cells into the gastric serosa of C57BL/6 mice. Tumor regions are outlined by black dotted lines (n = 5, per group).

u) Quantification of relative tumor area and tumor weight in orthotopic allografts derived from

ATK-transfected cells (n = 5, per group).

v and **w**) Flow cytometry gating strategy used to identify tumor-infiltrating immune cell subsets in gastric orthotopic allografts.

x) Percentage distribution of tumor-infiltrating immune cells in orthotopic allograft tumors (n = 5, per group).

y) Representative images of H&E and Ki-67 staining in gastric orthotopic allograft tumors. Scale bar, 100 μm.

z) Quantitative analysis of Ki-67-positive cells in orthotopic tumors derived from ATK-transfected cells. Each point represents an individual subject. All data in the statistical plots are shown as mean ± SD. Statistical significance is indicated by no significant (ns), *p < 0.05, **p < 0.01, **p < 0.001. Statistical analysis was performed using the Student's t-test (**c**, **o**, **u**, **x**, and **z**) and one-way ANOVA followed by Tukey's test (**f**, **g**, **h**, **j**, **k**, **l**, **n**, and **r**).

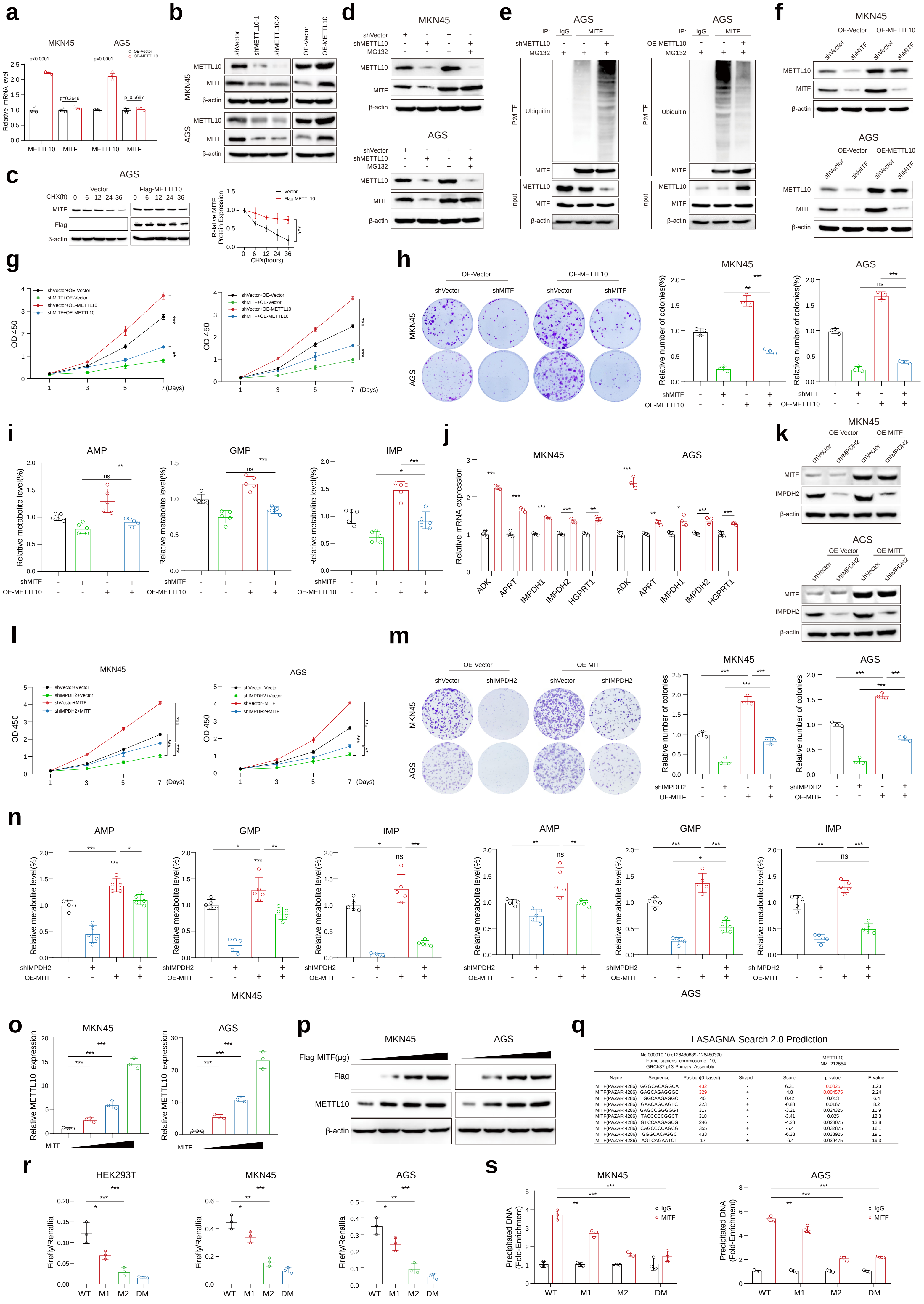

Nc 000010.10:c126480889-126480390 Homo sapiens chromosome 10, GRCh37.p13 Primary Assembly				METTL10 NM_212554		
Name	Sequence	Position(0-based)	Strand	Score	p-value	E-value
MITF(PAZAR 4286)	GGGCACAGGCA	432	-	6.31	0.0025	1.23
MITF(PAZAR 4286)	GAGCAGAGGGC	329	+	4.8	0.004575	2.24
MITF(PAZAR 4286)	TGGCAAGAGGC	46	-	0.42	0.013	6.4
MITF(PAZAR 4286)	GAACAGCAGTC	223	+	-0.88	0.0167	8.2
MITF(PAZAR 4286)	GAGCCGGGGGT	317	+	-3.21	0.024325	11.9
MITF(PAZAR 4286)	TACCCCCGGCT	318	-	-3.41	0.025	12.3
MITF(PAZAR 4286)	GTCCAAGAGCG	246	-	-4.28	0.028075	13.8
MITF(PAZAR 4286)	CAGCCCCAGCG	355	+	-5.4	0.032875	16.1
MITF(PAZAR 4286)	GGGCACAGGC	433	-	-6.33	0.038925	19.1
MITF(PAZAR 4286)	AGTCAGAATCT	17	+	-6.4	0.039475	19.3

Figure S2. METTL10 drives MITF activation and promotes nucleotide metabolism in gastric cancer cells

a) RT-qCPR of METTL10 and MITF protein levels in MKN45 and AGS cells with METTL10 overexpression.

b) Immunoblot analysis of METTL10 and MITF protein levels in MKN45 and AGS cells with METTL10 knockdown or overexpression.

c) MITF protein stability in Flag-METTL10 transfected-AGS cells treated with cycloheximide (CHX) for the indicated time points (top), and corresponding quantification of MITF levels relative to β-actin (bottom) (n = 3, independent experiments).

d) Immunoblotting of MITF in METTL10-knockdown MKN45 and AGS cells treated with or without MG132 (5 μM) for 5 hours.

e) Ubiquitination of MITF in AGS cells with METTL10 knockdown or overexpression , as assessed by immunoprecipitation and immunoblotting.

f) Immunoblot analysis of MITF expression in MKN45 and AGS cells co-transfected with shVector + Vector, shMITF + Vector, shVector + METTL10, or shMITF + METTL10 plasmids.

g) Cell proliferation curves of MKN45- and AGS-transfected cells by Cell Counting Kit-8 (CCK-8) assay (n = 3, independent experiments).

h) Colony formation assay of MKN45- and AGS-transfected cells (n = 3, independent experiments).

i) Quantification of purine metabolite levels in METTL10-transfected MKN45 cells (n = 5, per group).

j) mRNA expression levels of key purine metabolic enzymes in MKN45 and AGS cells transfected with MITF overexpression plasmids (n = 3, independent experiments).

k) Immunoblot analysis of IMPDH2 expression in MKN45 and AGS cells co-transfected with shVector + Vector, shIMPDH2 + Vector, shVector + MITF, or shIMPDH2 + MITF plasmids.

l) Cell proliferation curves of MKN45- and AGS-transfected cells by Cell Counting Kit-8 (CCK-8) assay (n=3, independent experiments).

m) Colony formation assay of MKN45- and AGS-transfected cells. (n=3, independent experiments).

n) Quantification of purine metabolite levels in MKN45- and AGS-transfected cells (n=5, per group).

o) Relative METTL10 mRNA levels in MKN45 and AGS cells transfected with increasing doses of MITF plasmids (n = 3, independent experiments).

p) METTL10 protein levels in MKN45 and AGS cells transfected with increasing doses of MITF plasmids.

q)In silico prediction of potential MITF binding sites within the METTL10 promoter using LASAGNA-Search 2.0. The two highest-confidence sites are highlighted in red. Promoter constructs include

mutation 1 (M1: deletion of GGGCACAGGCA), mutation 2 (M2: deletion of GAGCAGAGGGC), and double mutation (DM: M1 + M2).

r) Dual-luciferase reporter assay of METTL10 promoter activity in HEK293T, MKN45, and AGS cells transfected with Flag-MITF and promoter mutants M1, M2, or DM (n = 3, independent experiments).

s) Chromatin immunoprecipitation (ChIP)-qPCR assay showing direct binding of MITF to the METTL10 promoter in MKN45 and AGS cells transfected with Flag-MITF and M1, M2 or DM (n = 3, independent experiments). Each point represents an individual subject. All data in the statistical plots are shown as mean ± SD. Statistical significance is indicated by no significant (ns), *p < 0.05, **p < 0.01, ***p < 0.001. Statistical analysis was performed using the Student-t test (**c**, **j**, **o**, **r**, and **s**) and one-way ANOVA followed by Tukey's test (**g**, **h**, **i**, **l**, **m**, and **n**).

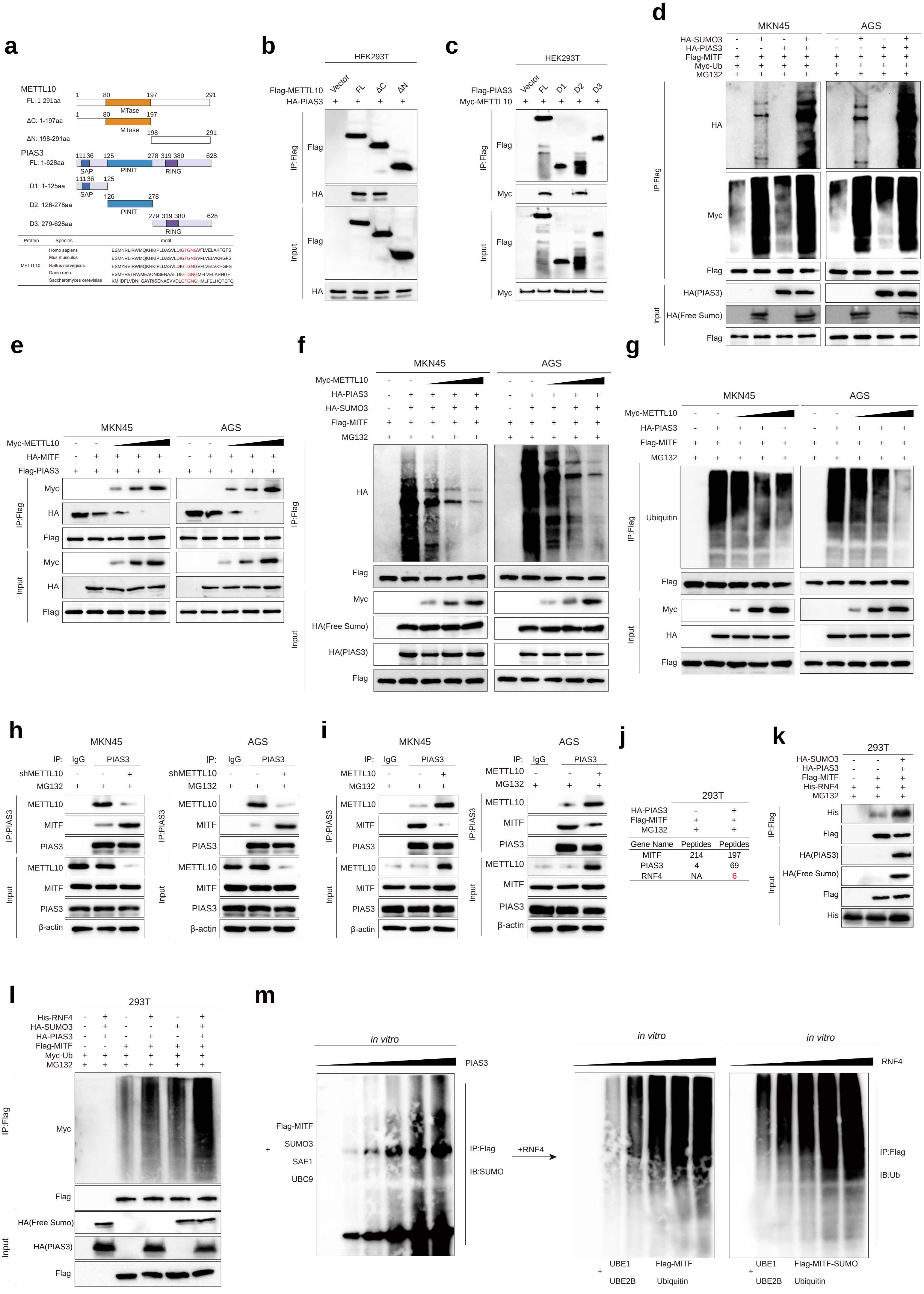
a
METTL10
FL: 1-291aa
ΔC: 1-197aa
ΔN: 198-291aa
MTase
PIAS3
FL: 1-628aa
D1: 1-125aa
D2: 126-278aa
D3: 279-628aa
SAP
PINIT
RING
Protein
Species
motif
METTL10
Homo sapiens
Mus musculus
Rattus norvegicus
Danio rerio
Saccharomyces cerevisiae
b
HEK293T
Flag-METTL10
HA-PIAS3
Vector
FL
ΔC
ΔN
IP:Flag
Input
Flag
HA
c
HEK293T
Flag-PIAS3
Myc-METTL10
Vector
FL
D1
D2
D3
Myc
d
MKN45
AGS
HA-SUMO3
HA-PIAS3
Flag-MITF
Myc-Ub
MG132
HA(PIAS3)
HA(Free Sumo)
e
Myc-METTL10
HA-MITF
Flag-PIAS3
f
g
Ubiquitin
h
IP:
IgG
PIAS3
shMETTL10
IP:PIAS3
METTL10
MITF
β-actin
i
j
293T
Gene Name
Peptides
MITF
214
197
PIAS3
4
69
RNF4
NA
6
k
His-RNF4
His
l
m
in vitro
Flag-MITF
SUMO3
SAE1
UBC9
IP:Flag
IB:SUMO
+RNF4
RNF4
IB:Ub
UBE1
UBE2B
Ubiquitin
Flag-MITF-SUMO

Figure S3. METTL10 competes with PIAS3 for MITF binding and inhibits MITF ubiquitination

a) Schematic representation of the domain structures of METTL10 and PIAS3 truncation mutants (top), and evolutionary conservation analysis of METTL10 residues among various species (bottom). Conserved methyltransferase catalytic motifs are highlighted in red.

b) Immunoprecipitation and immunoblot analysis of the interaction between HA-tagged full-length PIAS3 and Flag-tagged METTL10 truncation mutants in HEK293T cells.

c) Immunoprecipitation and immunoblot analysis of the interaction between Myc-tagged full-length METTL10 and Flag-tagged PIAS3 truncation mutants in HEK293T cells.

d) Immunoprecipitation and immunoblot analysis of ubiquitination and SUMOylation levels of MITF in MKN45 and AGS cells co-transfected with Myc-Ub, Flag-MITF, HA-SUMO3, and HA-PIAS3. Cells were pre-treated with the proteasome inhibitor MG132 (5 μM) for 5 hours prior to harvest.

e) Immunoprecipitation and immunoblot analysis of exogenous METTL10 or MITF binding to PIAS3 in MKN45 and AGS cells co-transfected with Myc-METTL10, HA-MITF, and Flag-PIAS3.

f) Immunoprecipitation and immunoblot analysis of MITF SUMOylation in MKN45 and AGS cells co-transfected with Myc-METTL10, HA-PIAS3, HA-SUMO3, and Flag-MITF. Cells were pre-treated with MG132 (5 μM) for 5 hours prior to harvest.

g) Immunoprecipitation and immunoblot analysis of MITF ubiquitination in MKN45 and AGS cells co-transfected with Myc-METTL10, HA-PIAS3, and Flag-MITF. Cells were pre-treated with MG132 (5 μM) for 5 hours prior to harvest.

h and **i**) Immunoprecipitation and immunoblot analysis of endogenous binding of METTL10 or MITF to PIAS3 in METTL10-knockdown (**h**) or overexpression (**i**) MKN45 and AGS cells. .

j) Mass spectrometry (LC-MS) identification of potential SUMO E3 ligases interacting with MITF in the presence or absence of PIAS3. Cells were pre-treated with MG132 (5 μM) for 5 hours prior to harvest.

k) Immunoprecipitation and immunoblot analysis of the interaction between MITF and RNF4 in HEK293T cells co-transfected with or without His-RNF4, Flag-MITF, HA-PIAS3, and HA-SUMO3. Cells were pre-treated with MG132 (5 μM) for 5 hours prior to harvest.

l) Immunoprecipitation and immunoblot analysis of MITF ubiquitination in HEK293T cells transfected with Myc-Ub, Flag-MITF, HA-SUMO3, and His-RNF4. Cells were pre-treated with MG132 (5 μM) for 5 hours prior to harvest.

m) Immunoblot analysis of the SUMOylation and ubiquitination levels of purified Flag-MITF protein following *in vitro* incubation with increasing concentrations of GST-PIAS3 or RNF4.

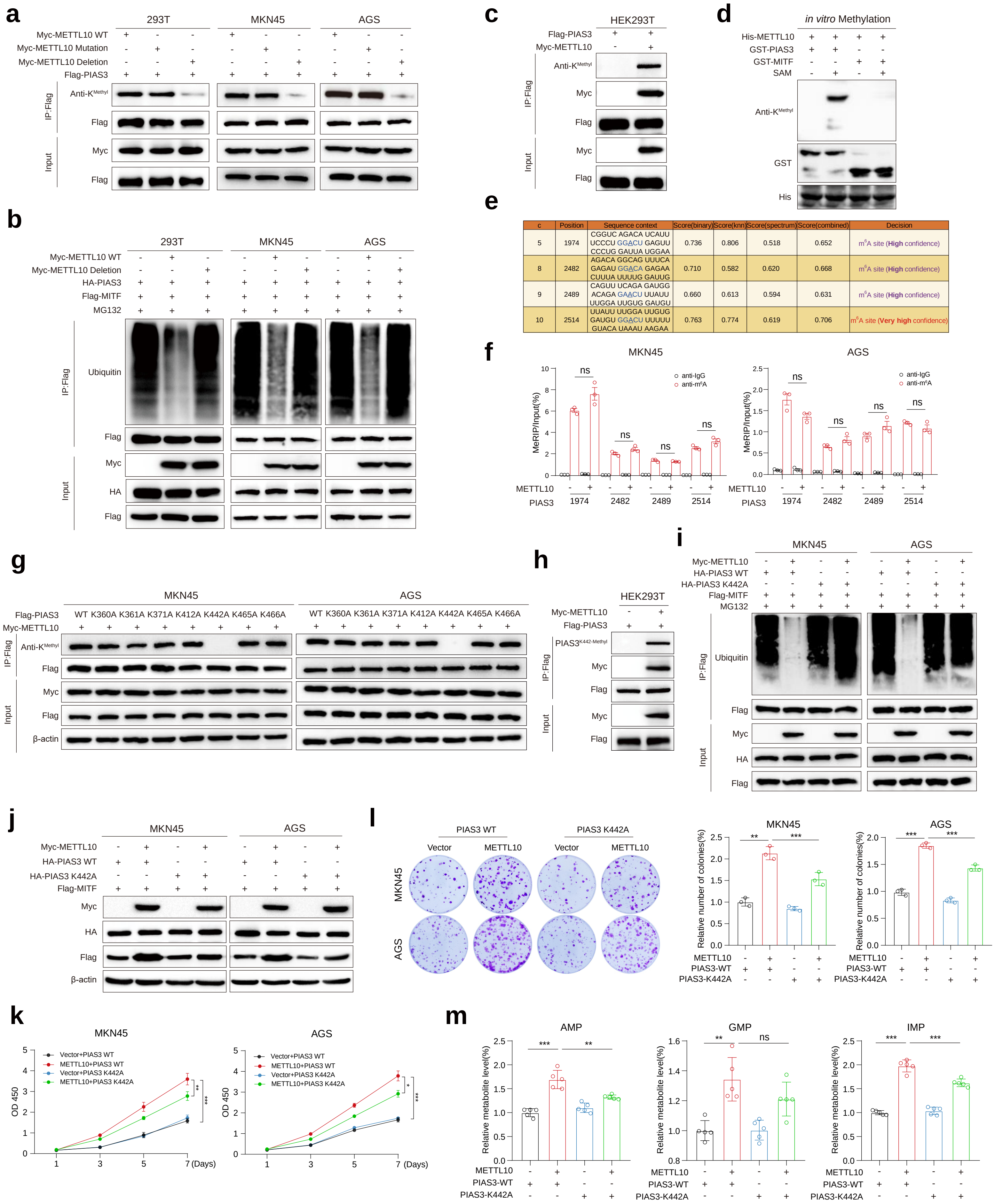

c	Position	Sequence context	Score(binary)	Score(knn)	Score(spectrum)	Score(combined)	Decision
5	1974	CGGUC AGACA UCAUU UCCCU GGACU GAGUU CCCUG GAUUA UGGAA	0.736	0.806	0.518	0.652	m6A site (**High** confidence)
8	2482	AGACA GGCAG UUUCA GAGAU GGACA GAGAA CUUUA UUUUG GAUUG	0.710	0.582	0.620	0.668	m6A site (**High** confidence)
9	2489	CAGUU UCAGA GAUGG ACAGA GAACU UUAUU UUGGA UUGUG GAUGU	0.660	0.613	0.594	0.631	m6A site (**High** confidence)
10	2514	UUAUU UUGGA UUGUG GAUGU GGACU UUUUU GUACA UAAAU AAGAA	0.763	0.774	0.619	0.706	m6A site (**Very high** confidence)

Figure S4. METTL10 catalyzes protein methylation of PIAS3

a) Immunoprecipitation and immunoblot analysis of total lysine methylation (Anti-K^{Methyl}) on exogenous PIAS3 in HEK293T, MKN45, and AGS cells co-transfected with Flag-PIAS3 and either Myc-METTL10 wild-type (WT), Myc-METTL10 SAM-binding motif mutant (DXGXGXG→AXAXAXA), or Myc-METTL10 deletion mutant (Δ85–91AA).

b) Ubiquitination levels of exogenous MITF in HEK293T, MKN45, and AGS cells transfected with Flag-MITF, HA-PIAS3, and either Myc-METTL10 WT or Myc-METTL10 deletion mutant (Δ85–91AA).

c) Immunoprecipitation and immunoblot of total lysine methylation of exogenous PIAS3 in HEK293T cells co-transfected with Flag-METTL10 and HA-PIAS3 WT.

d) *In vitro* methylation assay showing total lysine methylation (anti-K^{Methyl}) of purified GST-PIAS3 or GST-MITF protein incubated with purified His-METTL10 in the presence or absence of S-adenosylmethionine (SAM, 20 μM).

e) In silico prediction of potential m^6A modification sites on PIAS3 mRNA by the SRAMP tool.

f) MeRIP-qPCR analysis validating the top four predicted m^6A sites on PIAS3 mRNA in MKN45 and AGS cells with METTL10 expression.

g) Immunoprecipitation and immunoblot analysis of total lysine methylation of PIAS3 mutants (K360A, K361A, K371A, K412A, K442A, K465A, K466A) and wild-type (WT) in MKN45 and AGS cells co-transfected with Myc-METTL10.

h) Immunoprecipitation and immunoblotting analysis of $PIAS3^{K442\text{-}Methyl}$ on exogenous PIAS3 in HEK293T cells transfected with Myc-METTL10.

i) Ubiquitination assay of exogenous MITF in MKN45 and AGS cells co-transfected with Flag-MITF, Myc-METTL10, and either HA-PIAS3 WT or HA-PIAS3 K442A. Cells were pre-treated with the proteasome inhibitor MG132 (5 μM) for 5 hours prior to harvest.

j) Immunoblotting analysis of MITF in MKN45 and AGS cells transfected with Flag-MITF, Myc-METTL10, HA-PIAS3 WT or HA-PIAS3 K442A.

k) Cell proliferation curves of MKN45- and AGS-transfected cells by Cell Counting Kit-8 (CCK-8) assay (n = 3, independent experiments).

l) Colony formation assay of MKN45- and AGS-transfected cells (n = 3, independent experiments).

m) Quantification of purine metabolite abundance in the MKN45-transfected cells (n=5, per group). Each point represents an individual subject. All data in the statistical plots are shown as mean ± SD. Statistical significance is indicated by no significant (ns), $*p < 0.05$, $**p < 0.01$, $***p < 0.001$. Statistical analysis was performed using the one-way ANOVA followed by Tukey's test (**f**, **k**, **l**, and **m**).

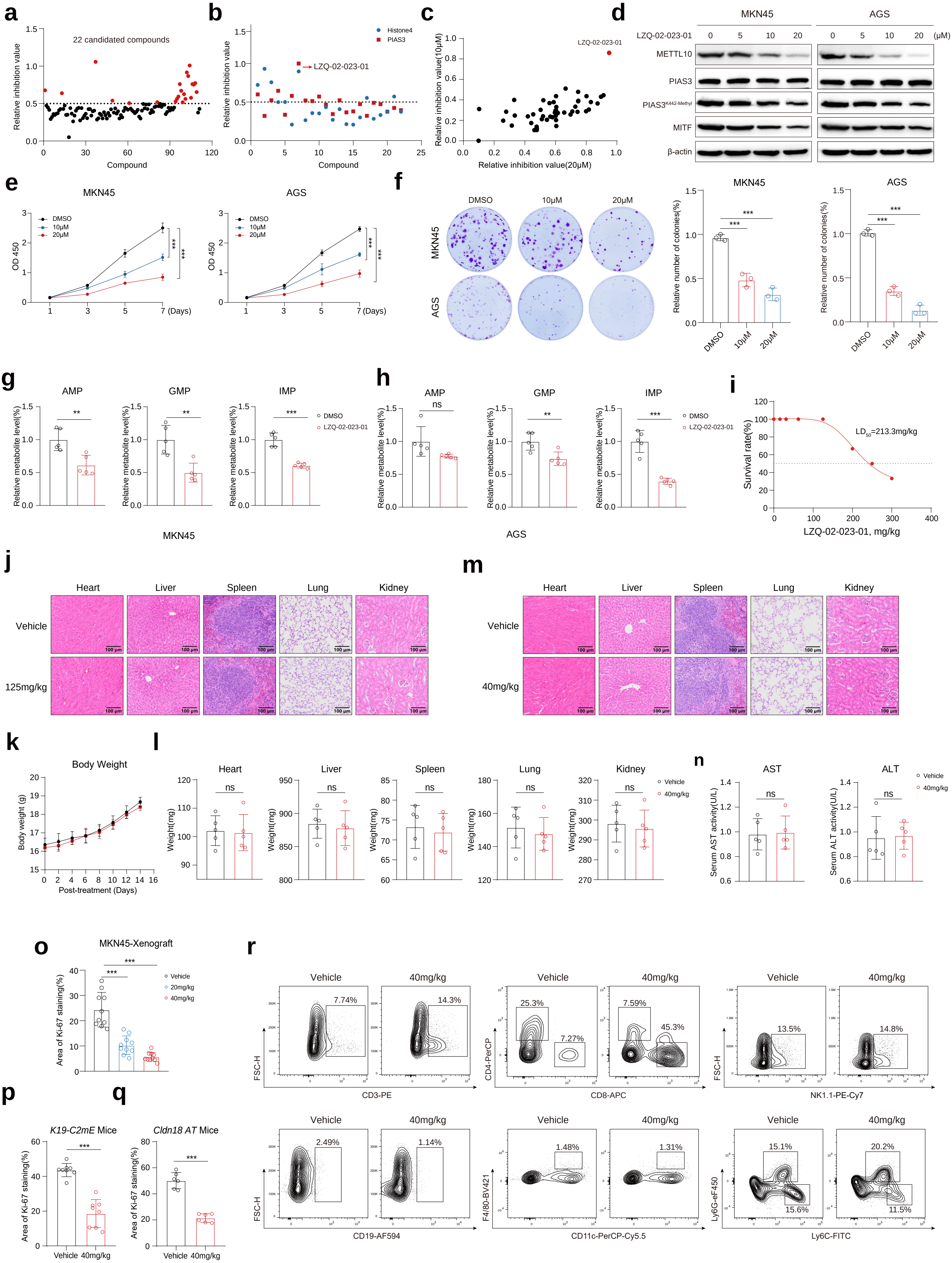

a
22 candidated compounds
Relative inhibition value
Compound
b
Histone4
PIAS3
LZQ-02-023-01
Relative inhibition value
Compound
c
LZQ-02-023-01
Relative inhibition value(10μM)
Relative inhibition value(20μM)
d
MKN45
AGS
LZQ-02-023-01
0 5 10 20
(μM)
METTL10
PIAS3
PIAS3K442-Methyl
MITF
β-actin
e
MKN45
AGS
DMSO
10μM
20μM
OD 450
(Days)
***
f
DMSO
10μM
20μM
MKN45
AGS
Relative number of colonies(%)
g
AMP
GMP
IMP
DMSO
LZQ-02-023-01
Relative metabolite level(%)
MKN45
h
AMP
GMP
IMP
ns
AGS
i
LD50=213.3mg/kg
Survival rate(%)
LZQ-02-023-01, mg/kg
j
Heart
Liver
Spleen
Lung
Kidney
Vehicle
125mg/kg
100 μm
m
Heart
Liver
Spleen
Lung
Kidney
Vehicle
40mg/kg
k
Body Weight
Body weight (g)
Post-treatment (Days)
l
Heart
Liver
Spleen
Lung
Kidney
Weight(mg)
Vehicle
40mg/kg
n
AST
ALT
Serum AST activity(U/L)
Serum ALT activity(U/L)
o
MKN45-Xenograft
Vehicle
20mg/kg
40mg/kg
Area of Ki-67 staining(%)
p
K19-C2mE Mice
q
Cldn18 AT Mice
Vehicle
40mg/kg
r
Vehicle
40mg/kg
7.74%
14.3%
CD3-PE
FSC-H
25.3%
7.27%
7.59%
45.3%
CD4-PerCP
CD8-APC
13.5%
14.8%
NK1.1-PE-Cy7
2.49%
1.14%
CD19-AF594
1.48%
1.31%
F4/80-BV421
CD11c-PerCP-Cy5.5
15.1%
15.6%
20.2%
11.5%
Ly6G-eF450
Ly6C-FITC

Figure S5. LZQ-02-023-01 is a selective and potent inhibitor of METTL10 with anti-tumor efficacy and favorable safety profile

a) A total of 111 small-molecule candidates with high predicted affinity for METTL10 were screened using a Histone 4 peptide as the substrate in an MTase-Glo assay. Among them, 22 compounds exhibited >50% inhibition of METTL10 enzymatic activity at 10 μM.

b) MTase-Glo assay identified LZQ-02-023-01 as the most potent inhibitor among the 22 compounds(10 μM), showing the highest inhibitory activity on METTL10 using Histone 4 peptide and GST-PIAS3 as substrates.

c) Structure-activity relationship (SAR) analysis further confirmed LZQ-02-023-01 as the most effective among its structural analogs in inhibiting METTL10 activity toward GST-PIAS3.

d) Immunoblotting analysis of METTL10, PIAS3, $PIAS3^{K442\text{-}Methyl}$ and MITF in MKN45 and AGS cells following 24-hour treatment with increasing concentrations of LZQ-02-023-01.

e) Cell proliferation curves of MKN45 and AGS cells treated with different doses of LZQ-02-023-01 by Cell Counting Kit-8 (CCK-8) assay (n = 3, independent experiments).

f) Colony formation assay of MKN45 and AGS cells treated with different doses of LZQ-02-023-01 (n = 3, independent experiments).

g and **h**) Quantification of purine metabolite levels in the MKN45 and AGS cells treated with LZQ-02-023-01(20μM).

i) Determination of the median lethal dose (LD_{50}) of LZQ-02-023-01 in ICR mice (n = 6, per group) via non-linear regression.

j) Acute toxicity assessments in ICR mice treated with LZQ-02-023-01(125mg/kg/day).H&E staining of internal organs tissues from ICR mice for histopathological evaluations. Scale bar, 100 μm.

k-n) Chronic toxicity assessments in ICR mice treated with LZQ-02-023-01(40mg/kg/day), including body weight tracking, organ weight measurements, ALT/AST evaluations, and H&E staining of major internal organs. Scale bar, 100 μm.

o-q) Quantification of Ki-67^{+} proliferative cells in MKN45-derived xenografts, *K19-C2mE* mice and Cldn18-AT mice treated with vehicle control or LZQ-02-023-01(40mg/kg).

r) Gating strategy used to identify immune cell subsets in gastric orthotopic ATK allografts treated with vehicle or LZQ-02-023-01. Each point represents an individual subject. All data in the statistical plots are shown as mean ± SD. Statistical significance is indicated by no significant (ns), $^{**}p < 0.01$, $^{***}p < 0.001$. Statistical analysis was performed using the one-way ANOVA followed by Tukey's test (**e, f** and **o**) and Student's t-test (**g**, **h**, **l**, **n**, **p**, and **q**).

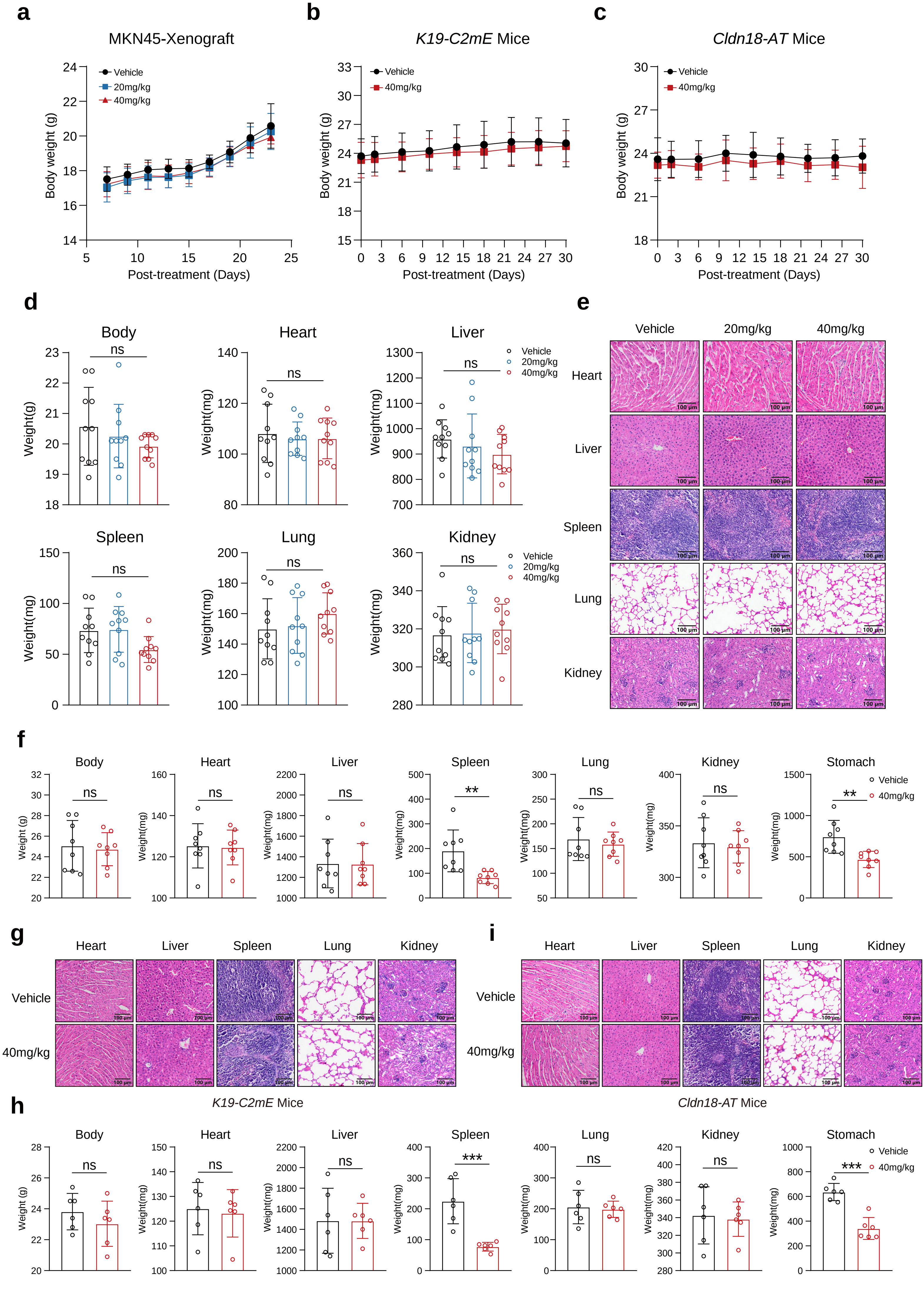

a
MKN45-Xenograft
Vehicle
20mg/kg
40mg/kg
Body weight (g)
Post-treatment (Days)
b
K19-C2mE Mice
Vehicle
40mg/kg
Body weight (g)
Post-treatment (Days)
c
Cldn18-AT Mice
Vehicle
40mg/kg
Body weight (g)
Post-treatment (Days)
d
Body
Heart
Liver
Spleen
Lung
Kidney
ns
Weight(g)
Weight(mg)
Vehicle
20mg/kg
40mg/kg
e
Vehicle
20mg/kg
40mg/kg
Heart
Liver
Spleen
Lung
Kidney
100 μm
f
Body
Heart
Liver
Spleen
Lung
Kidney
Stomach
ns
**
Weight (g)
Weight(mg)
Vehicle
40mg/kg
g
Heart
Liver
Spleen
Lung
Kidney
Vehicle
40mg/kg
K19-C2mE Mice
i
Heart
Liver
Spleen
Lung
Kidney
Vehicle
40mg/kg
Cldn18-AT Mice
h
Body
Heart
Liver
Spleen
Lung
Kidney
Stomach
ns
***
Weight (g)
Weight(mg)
Vehicle
40mg/kg

Figure S6. The small-molecule compound LZQ-02-023-01 exhibits a favorable safety profile

a-**c**) Body weight measurements of MKN45 xenograft mice (**a**), *K19-C2mE* mice (**b**) and *Cldn18-AT* mice (**c**) treated with LZQ-02-023-01 (20 mg/kg/day or 40 mg/kg/day).

d) Quantification of body and internal organ weights from MKN45 xenograft mice following LZQ-02-023-01 treatment.

e) Representative H&E staining of major internal organs from MKN45 xenograft mice treated with different doses of LZQ-02-023-01. Scale bar, 100 μm.

f) Quantification of body and internal organ weights from *K19-C2mE* mice treated with LZQ-02-023-01(40 mg/kg/day).

g) Representative H&E staining of major internal organs from *K19-C2mE* mice treated with of LZQ-02-023-01(40 mg/kg/day). Scale bar, 100 μm.

h) Quantification of body and internal organ weights from *Cldn18-AT* mice treated with LZQ-02-023-01(40 mg/kg/day).

i) Representative H&E staining of major internal organs from *Cldn18-AT* mice treated with treated with LZQ-02-023-01(40 mg/kg/day). Scale bar, 100 μm. Each point represents an individual subject. All data in the statistical plots are shown as mean ± SD. Statistical significance is indicated by no significant (ns), $^{**}p < 0.01$, $^{***}p < 0.001$. Statistical analysis was performed using the one-way ANOVA followed by Tukey's test (**d**) and Student's t-test (**f** and **h**).

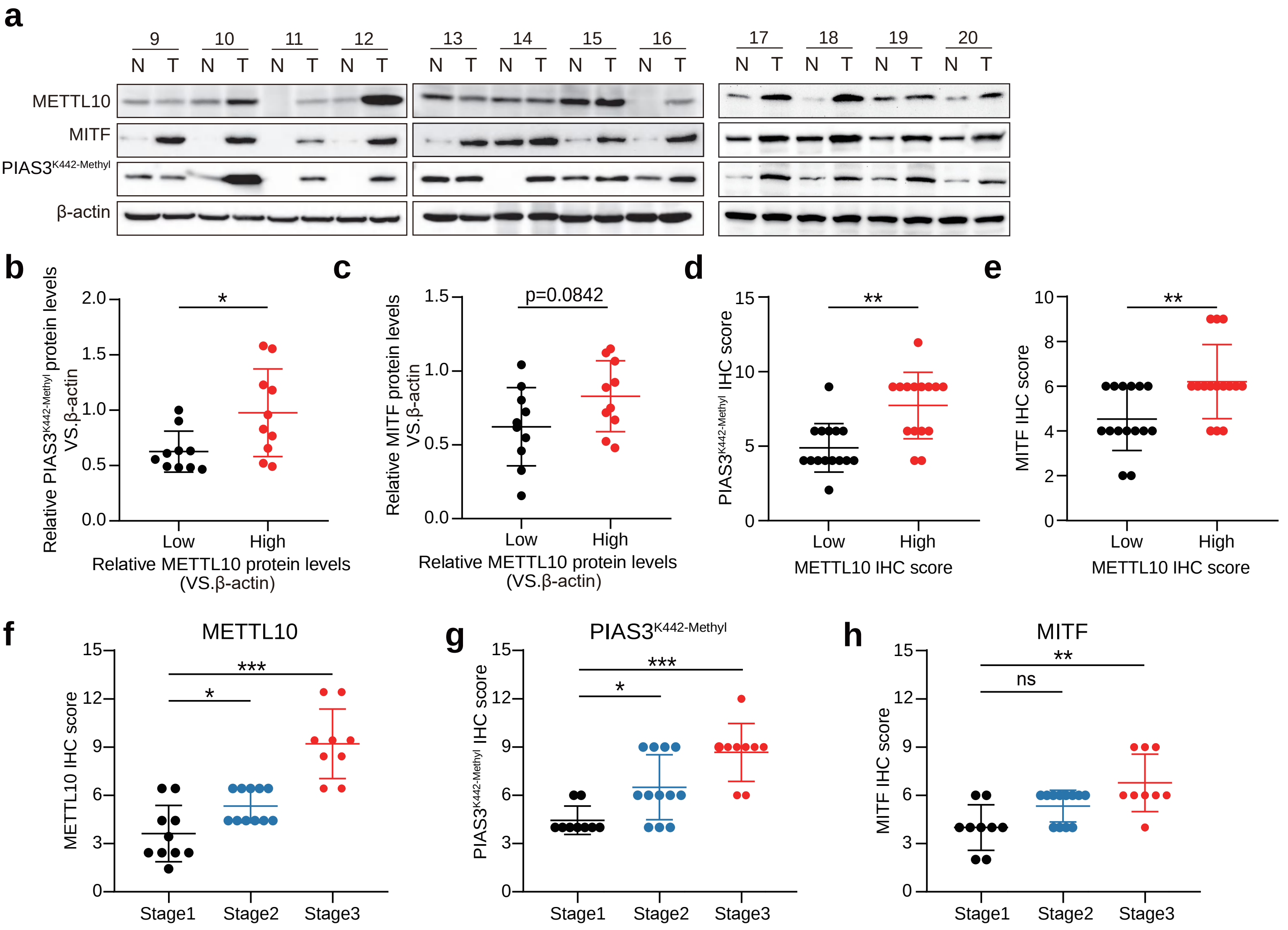

i

Gene expression of gastric cancer using multifactorial analysis with Stage

Variable	β	SE	Wald	P	OR	CI(2.5%)	CI(97.5%)
MITF	0.289	0.560	0.516	0.606	1.335	0.459	4.449
METTL10	1.128	0.567	1.989	0.047	3.089	1.146	11.305
PIAS3$^{K442\text{-}Methyl}$	1.342	0.569	2.360	0.018	3.827	1.521	14.796

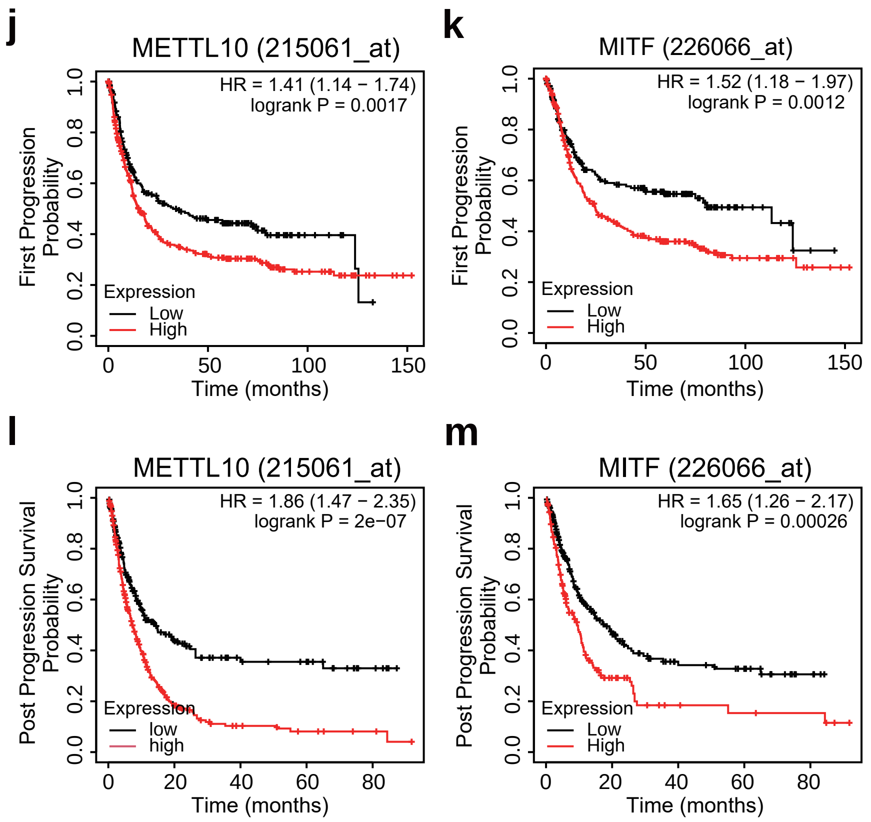

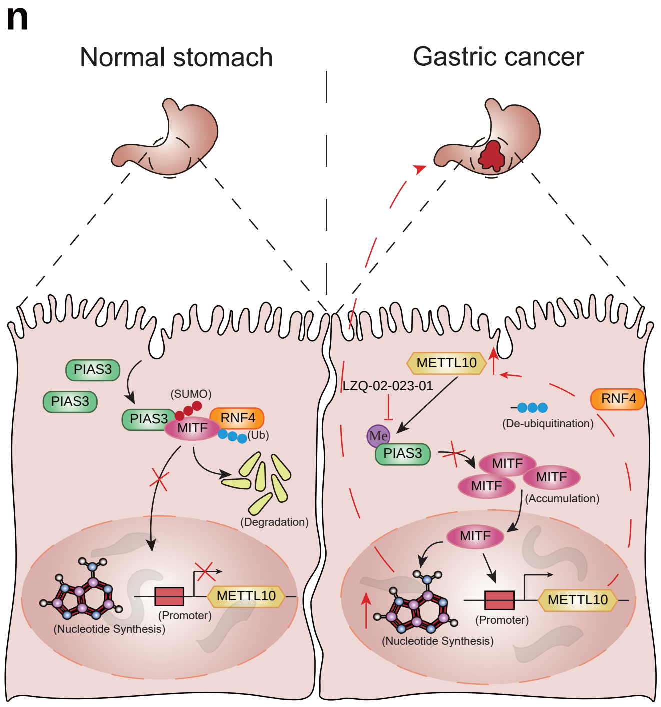

Figure S7. METTL10 and MITF expression levels exhibit clinical relevance in gastric cancer patients

a) Immunoblot analysis of METTL10, MITF, and PIAS3$^{K442\text{-}Methyl}$ in tumor tissues and matched adjacent normal tissues from 20 gastric cancer patients in our own cohort. An additional 8 paired samples are shown in Figure 7a.

b and **c**) Quantification of PIAS3$^{K442\text{-}Methyl}$ (**b**) and MITF (**c**) protein levels in tumor samples stratified by METTL10 expression level (low, n = 10; high, n = 10).

d and **e**) Quantification of immunohistochemical (IHC) staining scores for PIAS3$^{K442\text{-}Methyl}$ (**d**) and MITF (**e**) in tumor tissues with low (n = 15) or high (n = 15) METTL10 IHC scores.

f-h) IHC scores of METTL10 (**f**), PIAS3$^{K442\text{-}Methyl}$ (**g**), and MITF (**h**) across gastric cancer tissues of clinical stage I (n = 10), stage II (n = 11), and stage III (n = 9).

i) Multifactorial analysis evaluating METTL10, PIAS3$^{K442\text{-}Methyl}$, and MITF expression in our gastric cancer patient cohort (n = 30), adjusted for tumor stage.

j and **k**) Kaplan-Meier survival analysis of first progression (FP) for METTL10 (**j**) and MITF (**k**) in gastric cancer using the KM Plotter database.

l and **m**) Kaplan-Meier survival analysis of post-progression survival (PPS) for METTL10 (**l**) and MITF (**m**) in gastric cancer using the KM Plotter database.

n) Schematic model illustrating METTL10-mediated regulation of PIAS3/MITF signaling in gastric cancer progression. Each point represents an individual subject. All data in the statistical plots are shown as mean ± SD. Statistical significance is indicated by no significant (ns), *$p < 0.05$, **$p < 0.01$, ***$p < 0.001$. Statistical analysis was performed using the unpaired Student's t-test (**b**, **c**, **d**, and **e**), One-way ANOVA followed by Tukey's test (**f**, **g**, and **h**), Log-rank test (**j**, **k**, **l**, and **m**).
